# Supplementary material for: Genome-wide identification and characterization of the NF-Y proteins in Zanthoxylum armatum
Source: PeerJ. 2025 Jun 26;13:e19142. doi: 10.7717/peerj.19142 (PMC12206434; doi:10.7717/peerj.19142)
Supplement: Supplemental Information 6 [file peerj-13-19142-s006.docx]

**MIQE checklist**

**1 Experimental design**

**1.1 Definition of experimental and control groups**

Table 1 The expression of *ZaNF-Y* genes in different tissues of *Z. armatum*

| **Sampling name** | **Sampling period** |
| --- | --- |
| stems | flowering phase |
| leaves | flowering phase |
| female flowers | flowering phase |
| young fruits | 17 days after ovule initiation |

**1.2 Number within each group**

Different tissues of *Z. armatum* were collected from three plants.

**2 Sample**

**2.1 Description**

The different tissues (female flowers, young fruits, stems and leaves) of Z. armatum were collected from Juzi town in Leshan of Sichuan Province, China (29.32°N, 103.43°E). The plants of *Z. armatum* were cultivated in the standard greenhouse conditions; a 16-h-day/8-h-night cycle, 25℃ temperature, 60% relative humidity. Different tissues were haphazardly obtained from three plants and replicated three times. All samples were immediately frozen in liquid nitrogen and stored at -80 °C.

**2.2 If frozen, how and how quickly?**

All fresh plant samples were frozen in liquid nitrogen immediately and then stored at -80 °C.

**3 Nucleic acid extraction**

**3.1 Procedure**

1. Weigh an appropriate amount of plant sample ground with liquid nitrogen, add 500 μl of preheated 65°C Buffer PRL (please add 5% β-mercaptoethanol or 5% 2 M DTT before use), and immediately perform vigorous vortexing oscillation for 30-60 seconds to ensure complete lysis, reduce viscosity, and facilitate increased yield.
2. Incubate the lysate at 65°C in a water bath for 5 minutes, invert 1-2 times during the process to aid lysis, and then centrifuge at 12,000 rpm (13,400 × g) for 10 minutes.
3. Transfer the supernatant to a new 1.5 ml RNase-free centrifuge tube, add an equal volume of anhydrous ethanol (0.5 times the volume of the supernatant), and mix thoroughly by pipetting immediately.
4. Transfer the above mixture into the FastPure gDNA-Filter Column II (the FastPure gDNA-Filter Column II has already been placed in the collection tube), centrifuge at 12,000 rpm (13,400 × g) for 2 minutes, and discard the filtrate.
5. Place the FastPure gDNA-Filter Column II into a new 2 ml Collection Tube (provided in the kit), add 500 μl of Buffer PRLPlus, and centrifuge at 12,000 rpm (13,400 × g) for 30 seconds to collect the filtrate.
6. Add an amount of anhydrous ethanol equal to 0.5 times the volume of the filtrate, and immediately mix by aspiration to ensure homogeneity.
7. Transfer the above mixture to the FastPure RNA Column IV (which has already been placed in the collection tube), and centrifuge at 12,000 rpm (13,400 × g) for 2 minutes, then discard the filtrate.
8. Add 700 μl of Buffer PRW1 to the FastPure RNA Column IV, incubate at room temperature for 1 minute, then centrifuge at 12,000 rpm (13,400 × g) for 30 seconds, and discard the filtrate.
9. Add 500 µl of Buffer PRW2 (check if 48 ml of anhydrous ethanol has been added prior to use) to the FastPure RNA Column IV, centrifuge at 12,000 rpm (13,400 × g) for 30 seconds, and discard the filtrate.
10. Repeat step 9).
11. Return the FastPure RNA Column IV adsorption column to the collection tube, centrifuge at 12,000 rpm (13,400 × g) for 2 minutes to remove any residual Buffer PRW2 from the FastPure RNA Column IV.
12. Transfer the FastPure RNA Column IV to a new RNase-free 1.5 ml collection tube, and suspend-drop 30-100 µl of RNase-free ddH2O onto the center of the adsorption membrane. Incubate at room temperature for 2 minutes, then centrifuge at 12,000 rpm (13,400 × g) for 1 minute. The extracted RNA is stored at -80°C.

**3.2 Purity and concentration of RNA**

The Purity and concentration of RNA were assessed by NanoDrop Lite Spectrophotometer.

| Sample Name | concentration（ng/μL） | A260/A280 | A260/A230 |
| --- | --- | --- | --- |
| female flowers | 686 | 2.019 | 2.108 |
| young fruits | 567 | 2.13 | 2.257 |
| stems | 457 | 2.026 | 2.189 |
| leaves | 899 | 2.045 | 2.235 |

**3.3 RNA integrity: method**

The integrity of RNA were assessed using electrophoresis on 1.5% agarose gels.


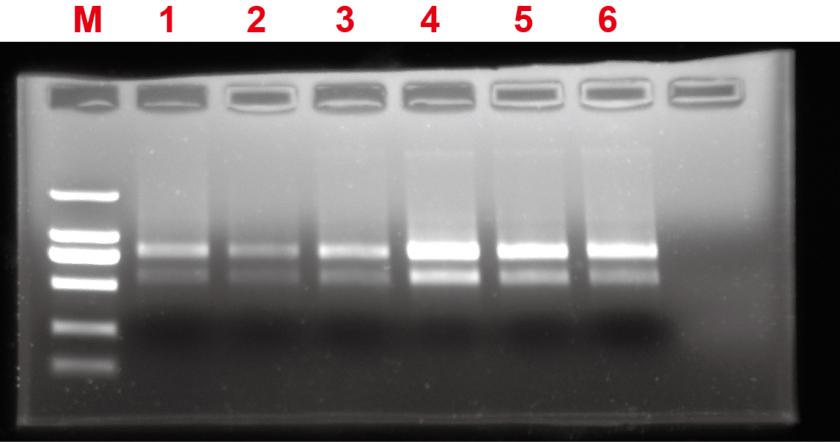


Fig. 1 1.5% agarose gel electrophoresis detection of total RNA extracted from different tissues

(M: 2000 bp DNA Marker; 1~6: Random selection of total RNA from different tissues)

**4 Reverse transcription**

**4.1 Complete reaction conditions**

Extracted RNA was reverse transcribed into cDNA using a ABScript III RT Master Mix for qPCR with gDNA Remover (ABclonal, RK20428). Operation steps refer to the manual.

**4.2 Amount of RNA and reaction volume**

Amount of RNA: 1 μg; reaction volume: 20µL.

**4.3 Priming oligonucleotide (if using GSP) and concentration**

Random Primers/Oligo d(T)_20_VN Primer Mix

**4.4 Reverse transcriptase and concentration**

5 X ABScript III RT Mix.

**4.5 Temperature and time**

| Temperature | Time |
| --- | --- |
| 37℃ | 2 min |
| 55℃ | 15 min |
| 85℃ | 5 min |
| 4℃ | Hold |

**5 qPCR target information and qPCR oligonucleotides**

| Gene symbol | Sequence accession number | Forward primer (5'->3') | Reverse primer (5'->3') | Amplicon length |
| --- | --- | --- | --- | --- |
| *ZaNF-YA1* | Zardc00391 | CATTCCACCGGGCACAGT | TTCCCCCAGCCAAAGCAG | 83 |
| *ZaNF-YA2* | Zardc02019 | CACCTCAAGTCGGGGCAG | CCATTGGTTGTCCCCCGT | 132 |
| *ZaNF-YA3* | Zardc03121 | AATGGCAATGGCAACGGC | CTTTCGCTTTGCTGCCCC | 119 |
| *ZaNF-YA4* | Zardc03815 | ACCCTCACCATCCGGGAT | CTTTCGCTTTGCTGCCCC | 91 |
| *ZaNF-YA5* | Zardc12046 | TATGCACCATTCGCGGCA | GGCTGAGGCTGTTGCTGA | 147 |
| *ZaNF-YA8* | Zardc23036 | TGTGCGTCGCATCCATGT | AAGGGGCAAAGGCATGGG | 114 |
| *ZaNF-YA9* | Zardc23708 | GCTCAGCCCAGCATCACA | AAGCCACTGGTGCCATGG | 82 |
| *ZaNF-YA11* | Zardc25782 | CCTGCCGGAGTTTGGGTT | GTGCAGGTTGCCATTGCC | 85 |
| *ZaNF-YA12* | Zardc27486 | GGGATCATCAGGCGTCGG | GTCCACCACACCCCCTTG | 130 |
| *ZaNF-YA15* | Zardc30673 | TCCAGCAGCAGGAGTTTGG | GGTGCAGGTTGCCATTGC | 102 |
| *ZaNF-YA16* | Zardc39364 | CAGGCAGGGTAATGGGGC | CGAGTTGGAGGGCATGCA | 116 |
| *ZaNF-YA17* | Zardc42135 | GTGCCGCTGCCTCTTGAA | GCTTCAAGCTTGGCACGG | 104 |
| *ZaNF-YA18* | Zardc47788 | TCAAGCAGCGGCAACAGA | TGGGTGCTTCTGCTGCTC | 147 |
| *ZaNF-YA19* | Zardc51606 | GAAGGGCTAGGGGTTGCG | TCCAGTGCCATCGGAGGA | 128 |
| *ZaNF-YA20* | Zardc55146 | GGGAAGCATGCGGAGGAG | CTTCGCCCGTCAGAGCAA | 102 |
| *ZaNF-YB1* | Zardc01252 | TGGGGAGGGAGAAGGACG | AAACCCCACCACCAGCAC | 117 |
| *ZaNF-YB5* | Zardc07335 | TTCGTCACCGGTGAAGCC | AAGAGGGAGAGGGGCTCG | 125 |
| *ZaNF-YB6* | Zardc07340 | ATAAGGACGTGCCGCCTC | CCCCCAGAGCCATACACG | 88 |
| *ZaNF-YB7* | Zardc08835 | CCAACGTTGGCCGGATAA | TCGCCTTTCCTTTCTGCACT | 146 |
| *ZaNF-YB8* | Zardc09563 | GAGCTCTGCCTGGCCAAA | TCATGTGCTGCCCCTGTG | 84 |
| *ZaNF-YB9* | Zardc16260 | TGAGACAAAACGGCGCCA | CTTTGCATTCGCCGGCAG | 118 |
| *ZaNF-YB10* | Zardc16329 | GGACCGAACAGCAGCGAT | CTTTGCATTCGCCGGCAG | 102 |
| *ZaNF-YB11* | Zardc16775 | CGCCAACCAATGCATCGG | TGCTGGAGCTCACATGCA | 103 |
| *ZaNF-YB12* | Zardc17774 | GGGTGGGGATGGATCTGC | GCGCACCCTGAAGAGCAT | 80 |
| *ZaNF-YB13* | Zardc17778 | AAGATCGCCAAGGACGCC | ATCACTTGCCTCGCTGGT | 81 |
| *ZaNF-YB15* | Zardc23890 | TAACGAGTCAGGTGGCGG | GCATTAGCAGGCAACGCT | 126 |
| *ZaNF-YB16* | Zardc27061 | CCTTCTCTCGCGCCCTTT | ACTTTGGTCGCCGCTCTC | 130 |
| *ZaNF-YB18* | Zardc30065 | CCCTAACCATGCTGCTGCT | TGGTACCTCCATTGCCACG | 88 |
| *ZaNF-YB19* | Zardc32065 | AATGGGGCCGCAATGACA | GGTTAGGGCCTGGTTGCT | 118 |
| *ZaNF-YB20* | Zardc34227 | TCCAATTGCCAACGTGGG | GACGCCTCACCCGTTACA | 129 |
| *ZaNF-YB21* | Zardc37513 | TGGAGGGTGAGACCAGGG | TTTGGCCAGGCAGAGCTC | 81 |
| *ZaNF-YB24* | Zardc44516 | GACTCGCTTGGGGCACAA | AAAGCTCCTGCCCCCAAA | 89 |
| *ZaNF-YB28* | Zardc50176 | GCAGGATTGCTCACCCGA | CGTGGCGGAGAGGTAGTG | 100 |
| *ZaNF-YB29* | Zardc50481 | TTCTCCAATCCTCGCGCC | TTGTTCGCGGACGTTCGA | 140 |
| *ZaNF-YC1* | Zardc03336 | AGCAGCAGCCTGTGATGG | GAGGGATGGCCATGGCA G | 108 |
| *ZaNF-YC2* | Zardc08780 | ATGTCCCACCGCAGCATC | CCAGGTCGAGGCTGTTGG | 97 |
| *ZaNF-YC4* | Zardc09836 | CCAAAGCCTGCGAGCTCT | TAATGGCGGCAGCGATGT | 108 |
| *ZaNF-YC5* | Zardc12609 | ATTTCTGGCCACCGTGCA | ATACACGGGTGCCATGCC | 135 |
| *ZaNF-YC6* | Zardc16762 | TGGCGGACAACCCACATC | GGAATTGCCCCAGCCGAT | 135 |
| *ZaNF-YC9* | Zardc32870 | CGGTTCCCATGGTGGCTT | GGTGAAGGTGCTGGGCTT | 120 |
| *ZaNF-YC10* | Zardc34600 | TGTCACATCTGCTGGGGC | TGCTGTTGCTGCTGGTGA | 105 |
| *ZaNF-YC11* | Zardc37300 | TACCCACCTCAGCCTGCA | CTTGGCGCTGGTAGGACC | 91 |
| *ZaNF-YC12* | Zardc37796 | TCTGGTGGTCCCAACCCT | CAGCTGAGCACCTCCTGG | 81 |
| *ZaNF-YC13* | Zardc37797 | AGCAAGGACATGGCGGAC | ACTGGTCCAGGGTTGGGA | 121 |
| *ZaNF-YC14* | Zardc38429 | GGGCCAGGTTACCAGCAG | ATGCTGTGGAGCCGTACG | 133 |
| *ZaNF-YC15* | Zardc38432 | GGGCCAGGTTACCAGCAG | GCAGTATGGGGTTCGGGC | 123 |
| *ZaNF-YC16* | Zardc39205 | CCCCAGCCTCCCTCTTCT | CTTGGCGCTGGTAGGACC | 109 |
| *ZaNF-YC17* | Zardc39938 | AAGGCAAACCGCAGTCCA | ACCTCGTCCTCGGACTCA | 80 |
| *ZaGAPDH* | Zardc45087 | TGGTGAGAAGCCCGTTGC | CAACGTACTCGGCTCCGG | 80 |

**6 qPCR protocol**

**6.1 Complete reaction conditions**

qRT-PCR was performed on QX96M quantitative PCR system (JLM, Chengdu, China) using a 2X Universal SYBR Green Fast qPCR Mix (ABclonal, RK21203).

**6.2 kit identity and manufacturer**

2X Universal SYBR Green Fast qPCR Mix (ABclonal, RK21203)

**6.3 Additives**

2X Universal SYBR Green Fast qPCR Mix (ABclonal, RK21203)

**6.3 Reaction volume and amount of cDNA/DNA**

| **component** | **volume** | **Final concentration** |
| --- | --- | --- |
| template | 2 µL | - |
| Forward primer(10µM) | 0.4 µL | 0.2 µM |
| Reverse primer(10µM) | 0.4 µL | 0.2 µM |
| 2X Universal SYBR Green Fast qPCR Mix | 10 μL | 2× |
| ddH_2_O | 7.2 µL | - |
| Total | 20 µL | - |

**6.4 Complete thermocycling parameters**

95 ◦C 3 min;

95 ◦C 5 s;

40 cycles

60 ◦C 30 s;

Dissociation stage

**7 qPCR validation**

**7.1 Specificity (gel, sequence, melt, or digest)**

*ZaNF-YA1
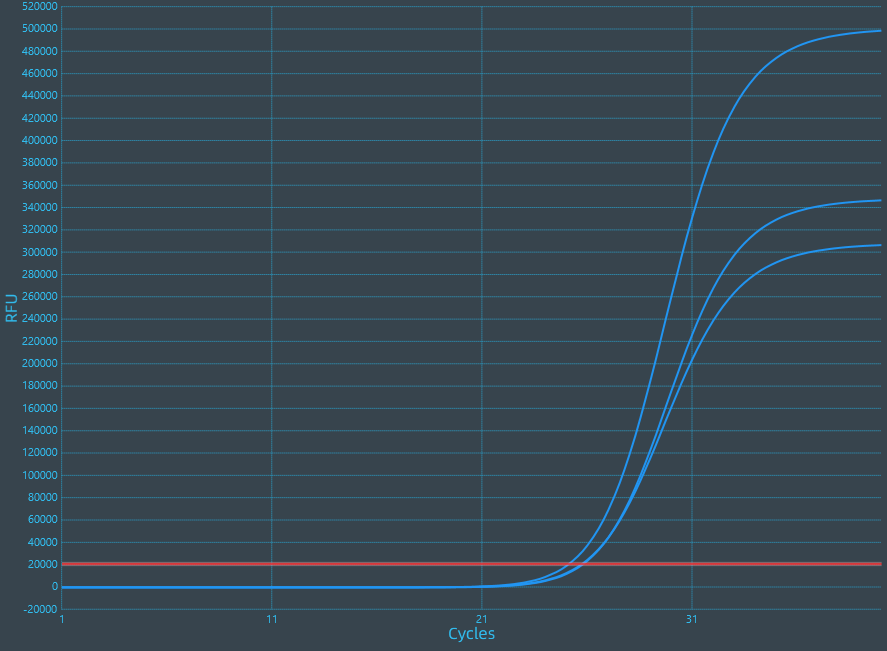

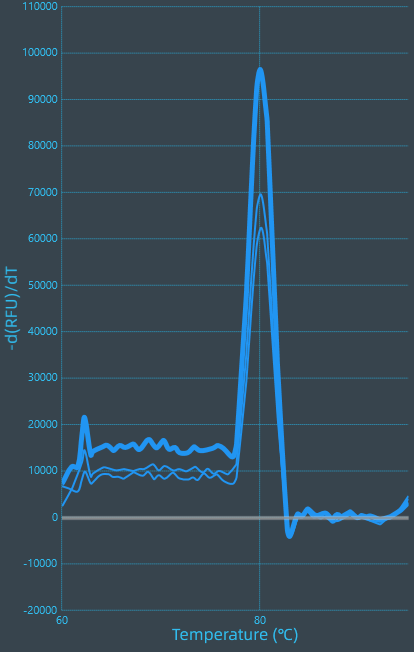
*

*ZaNF-YA2
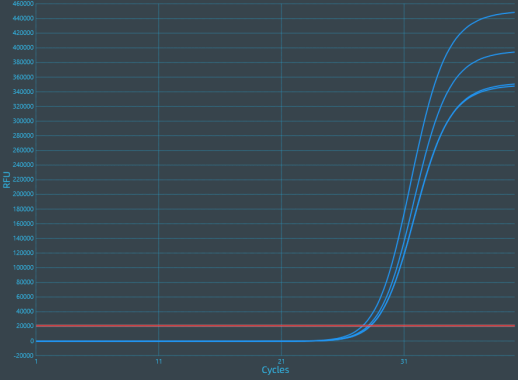

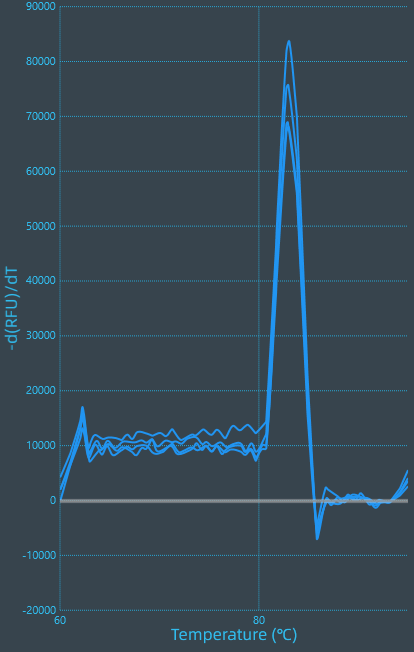
*

*ZaNF-YA3
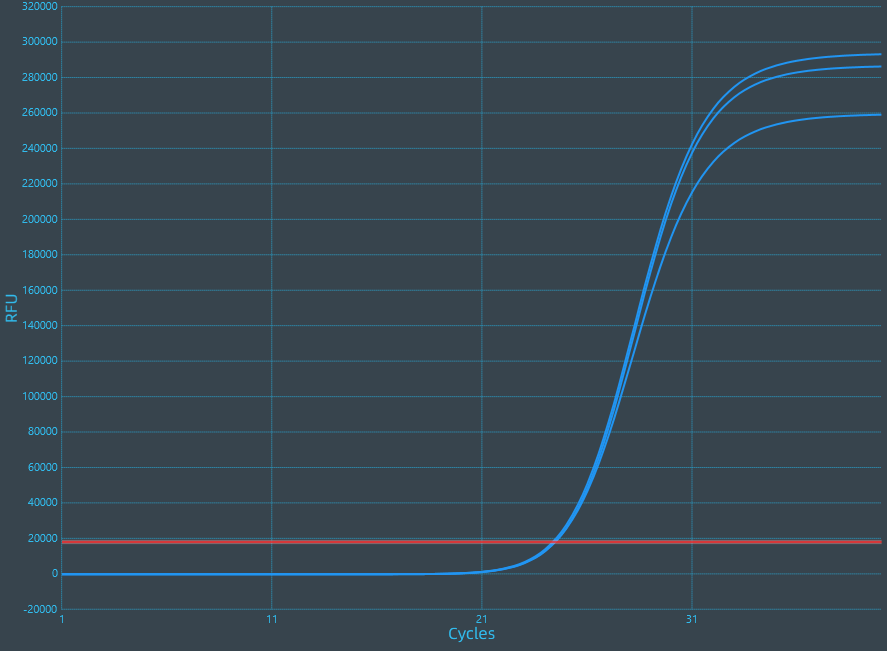

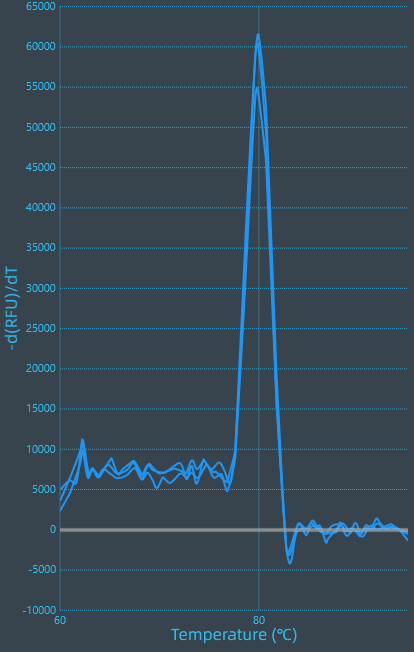
*

*ZaNF-YA4
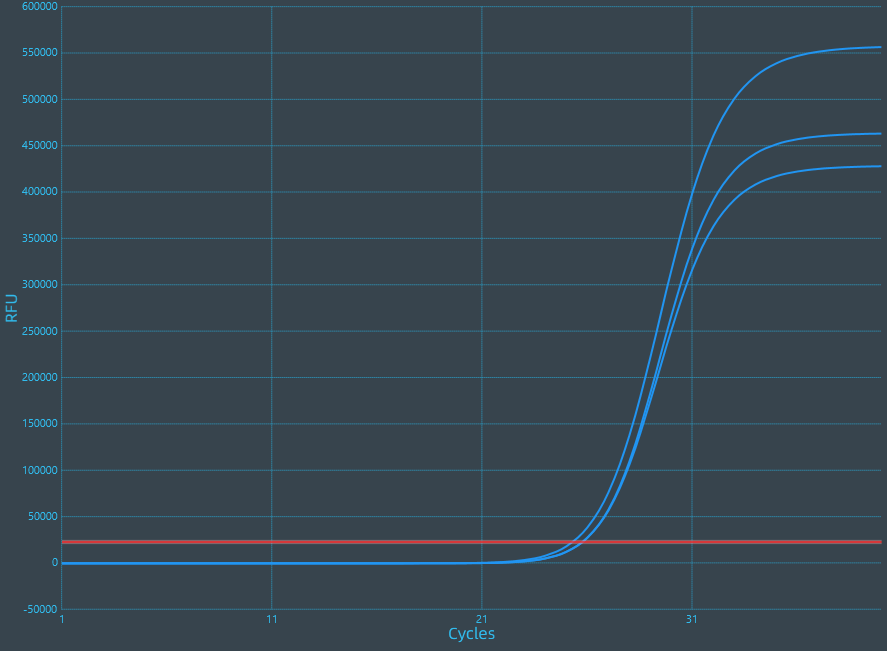

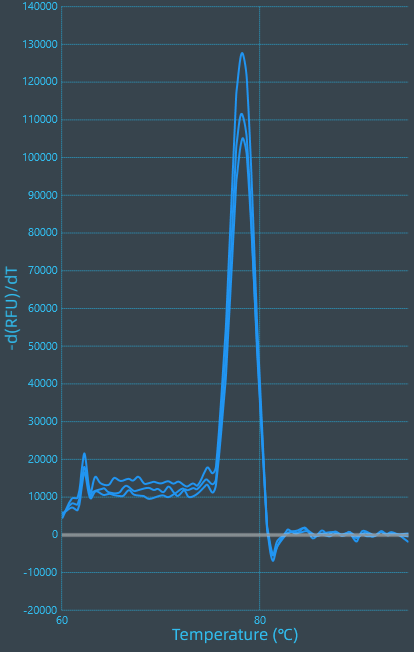
*

*ZaNF-YA5
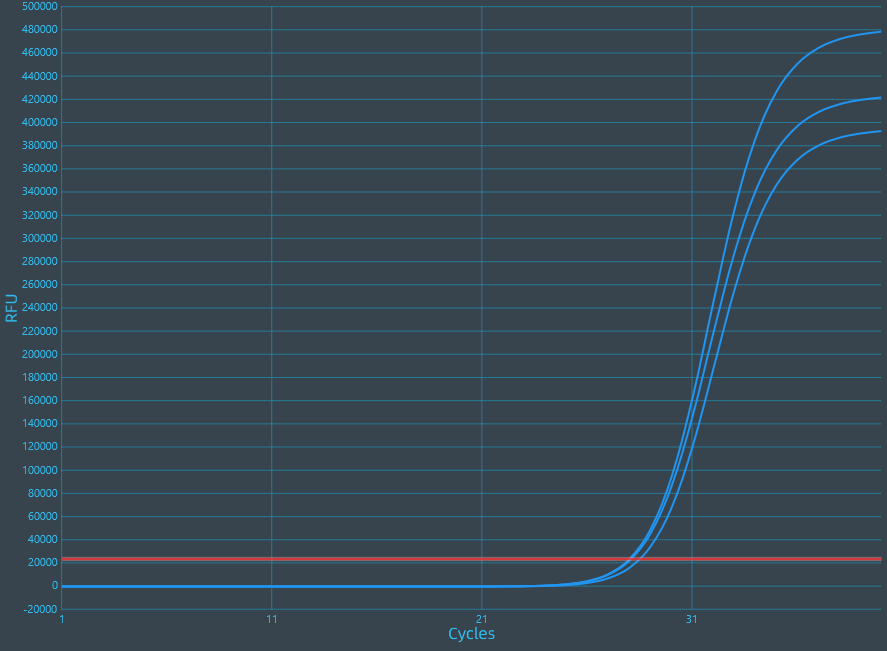

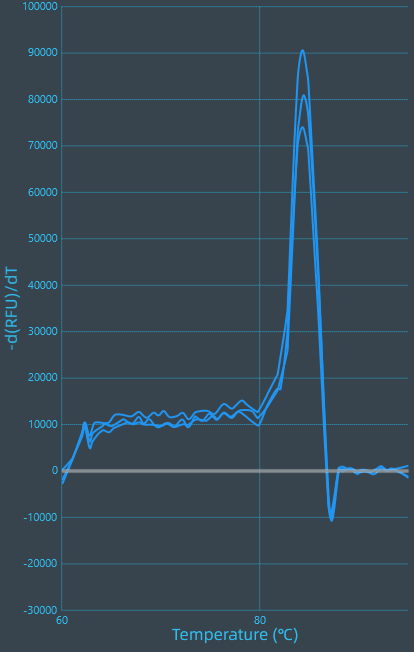
*

*ZaNF-YA8*
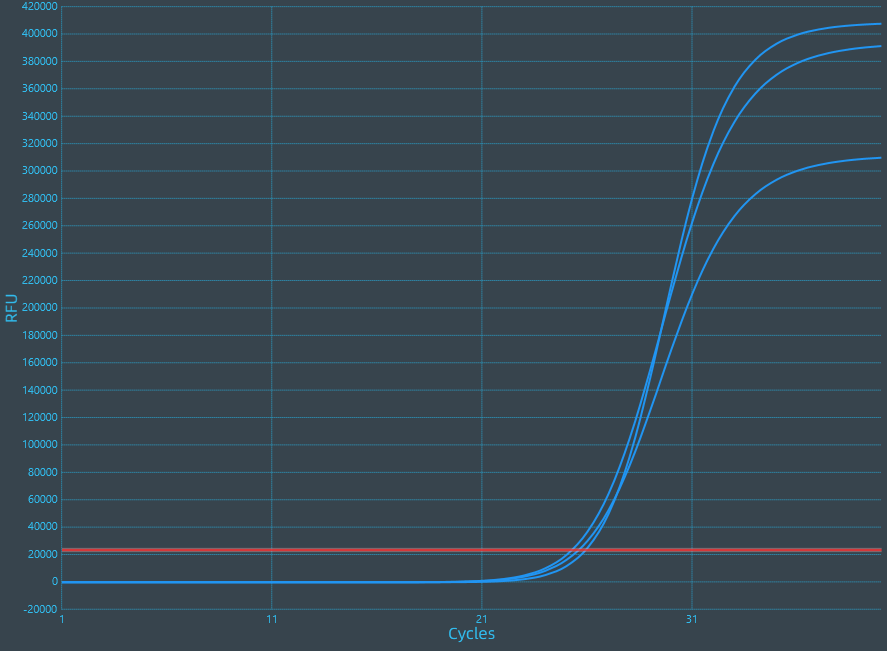

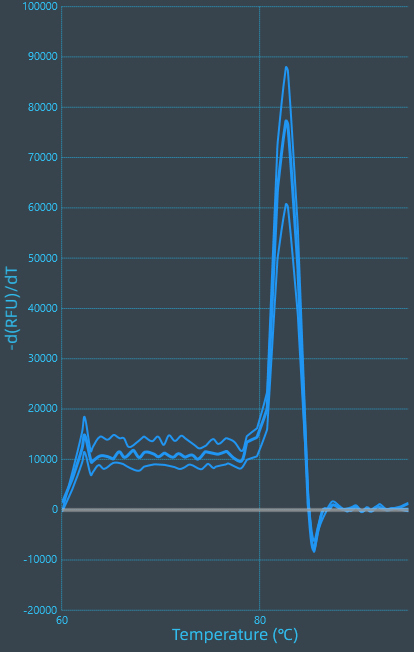


*ZaNF-YA9*
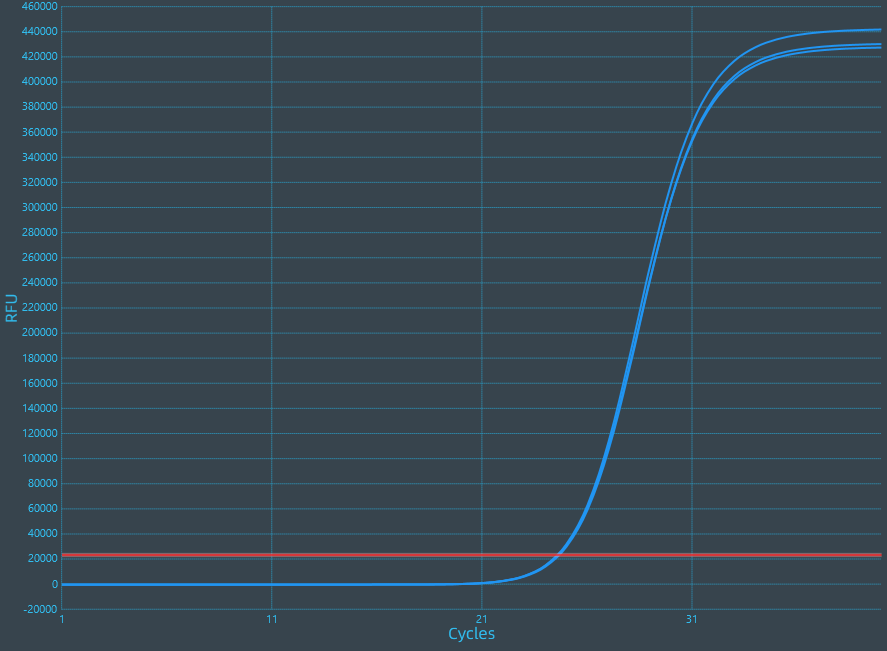

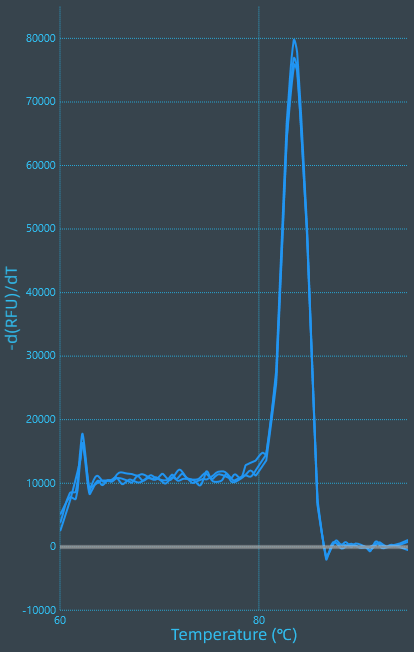


*ZaNF-YA11*
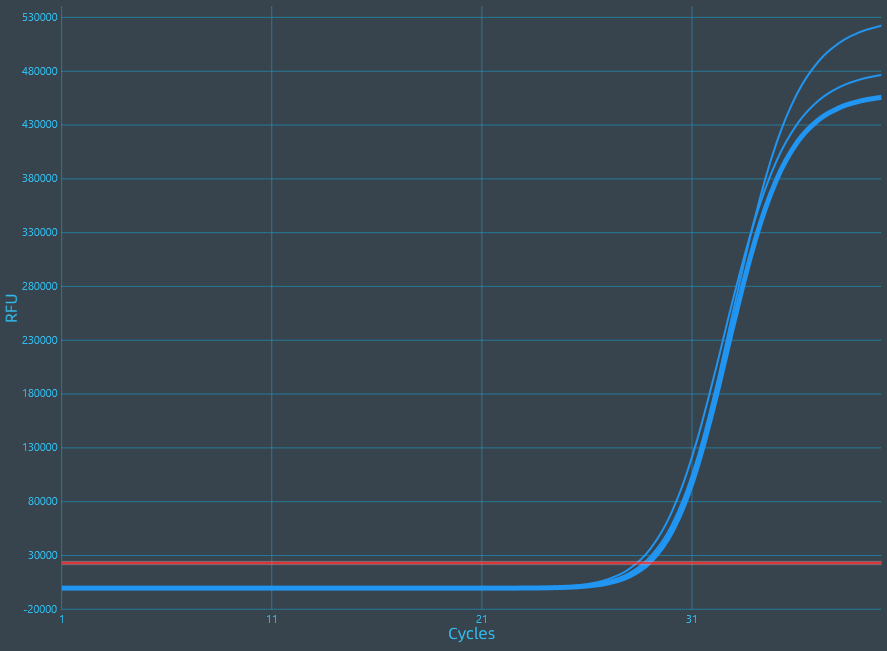

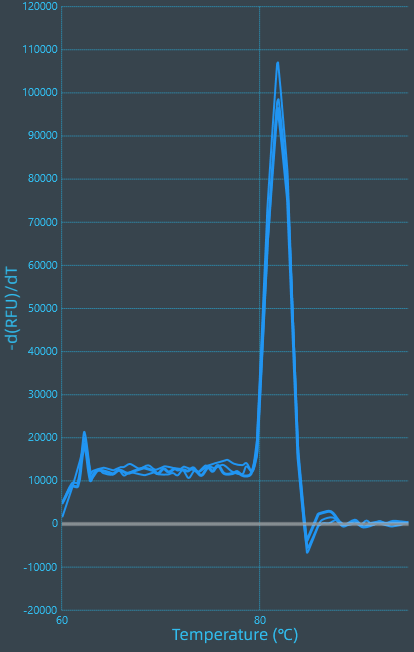


*ZaNF-YA12
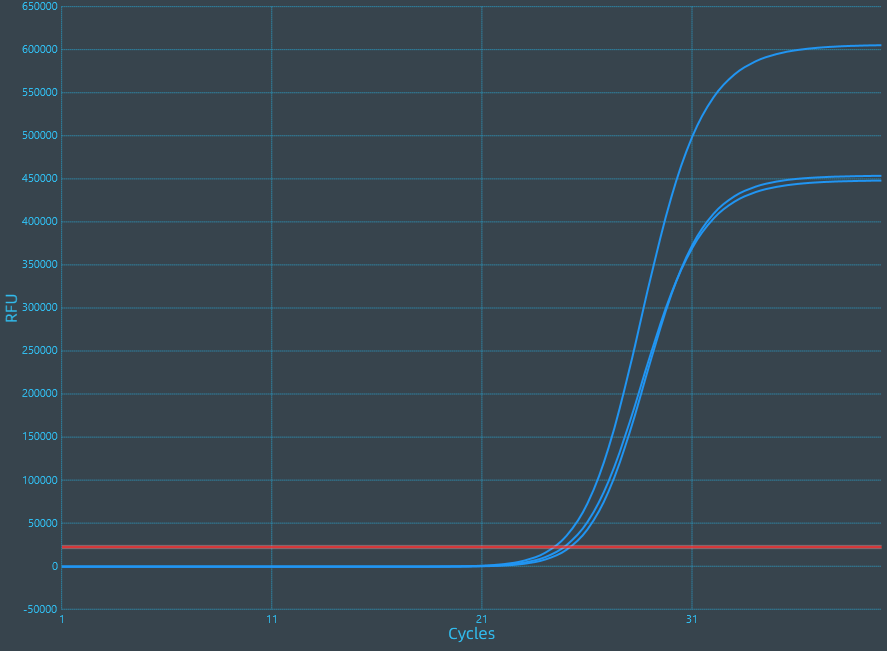

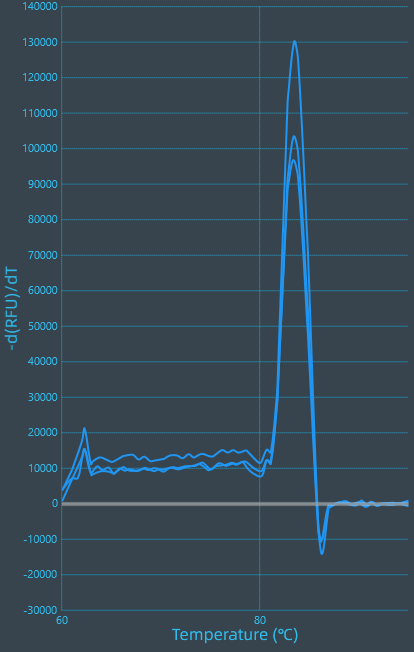
*

*ZaNF-YA15
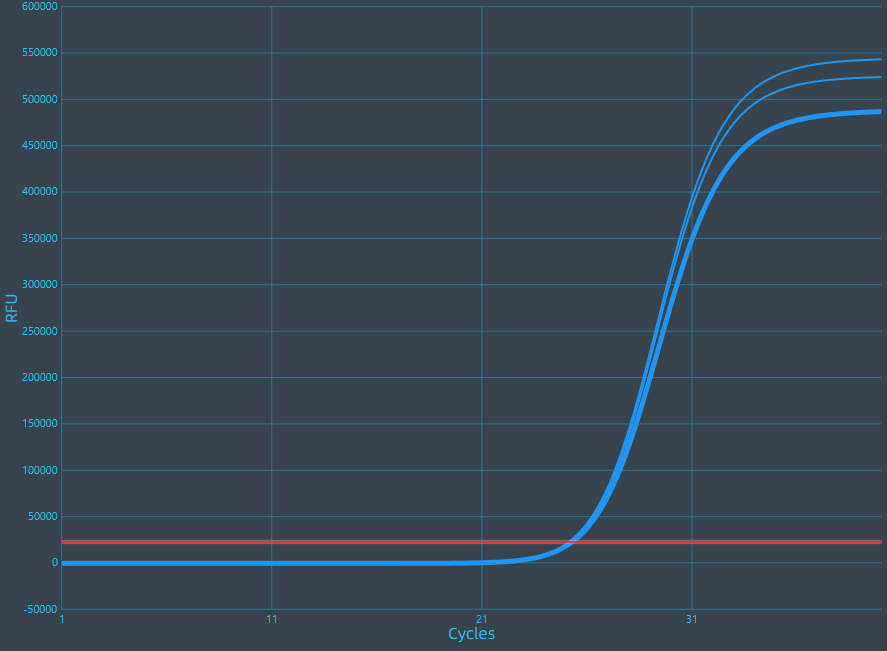

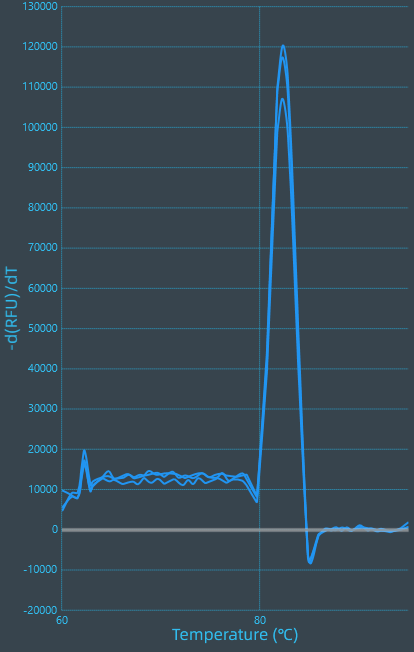
*

*ZaNF-YA16*
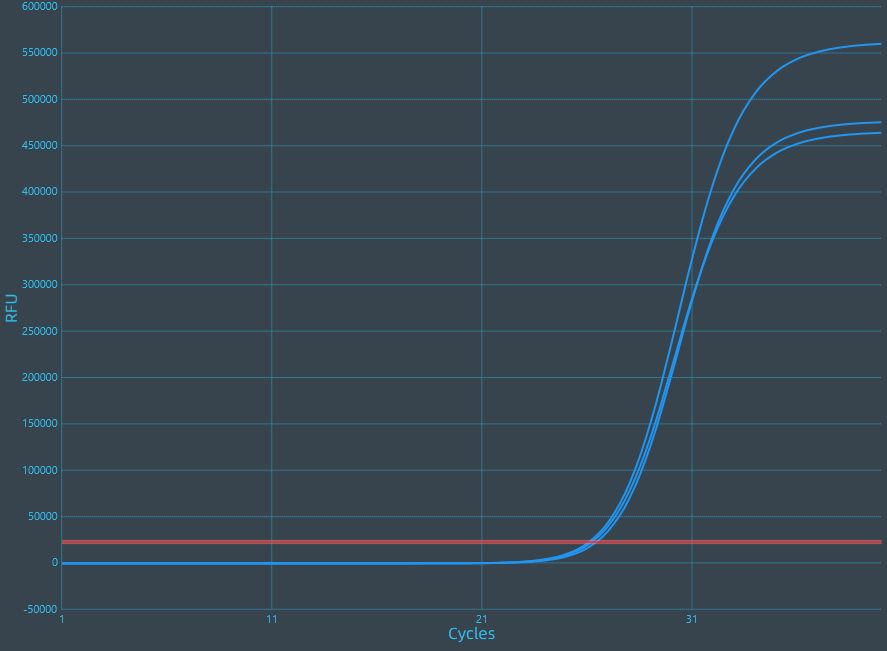

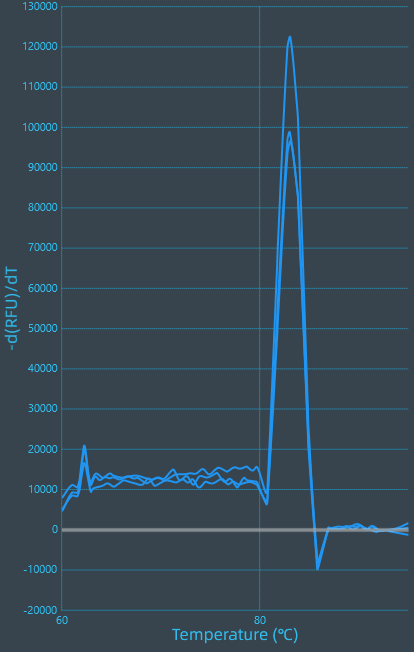


*ZaNF-YA17*
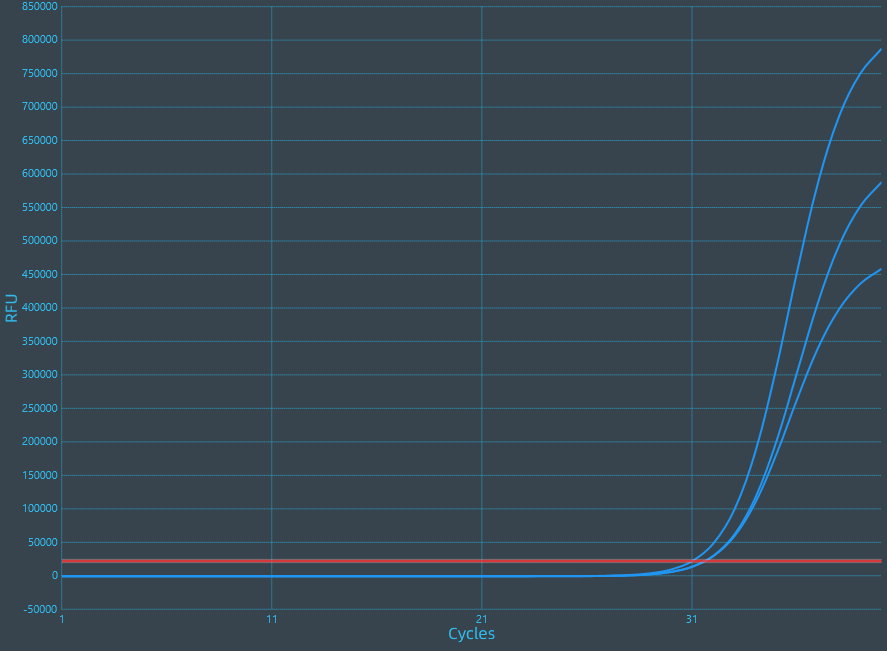

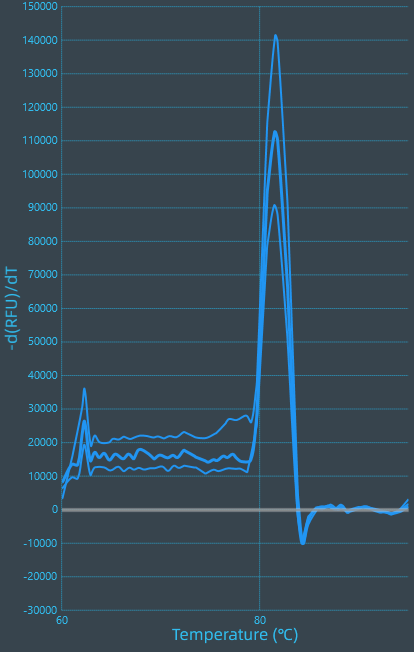


*ZaNF-YA18*
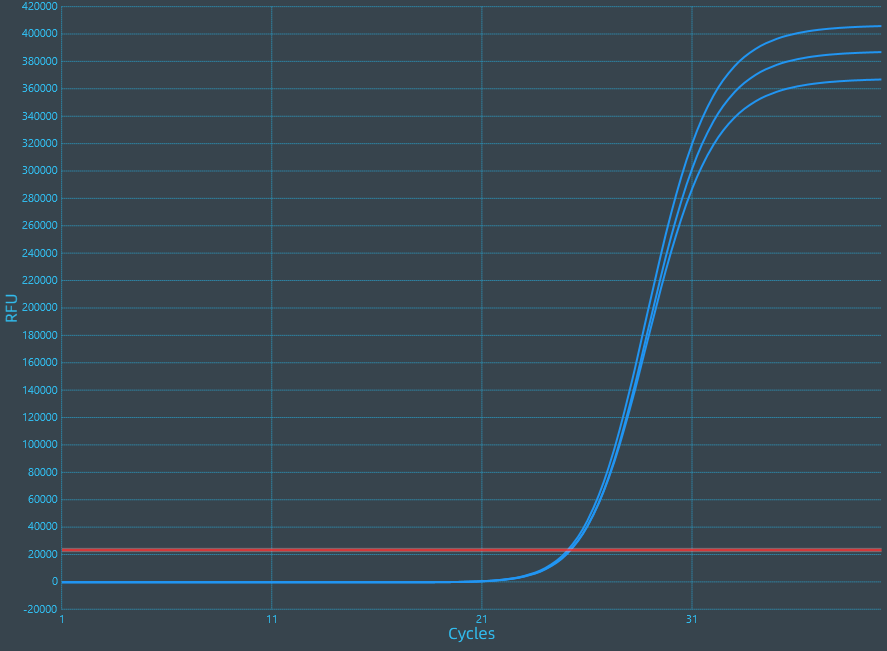

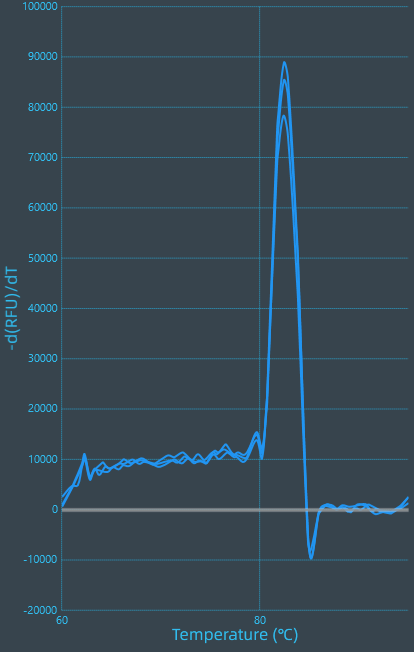


*ZaNF-YA19*
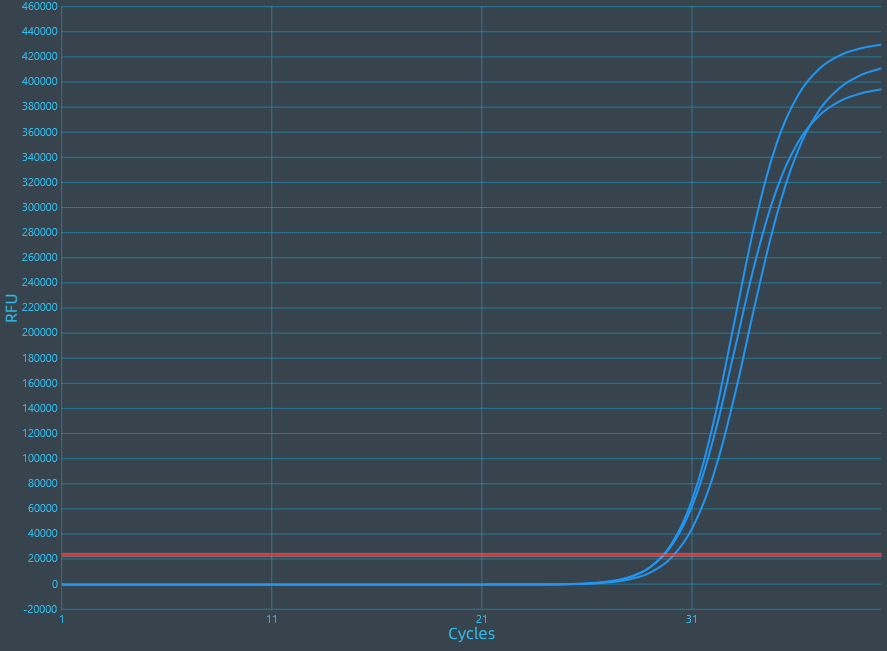

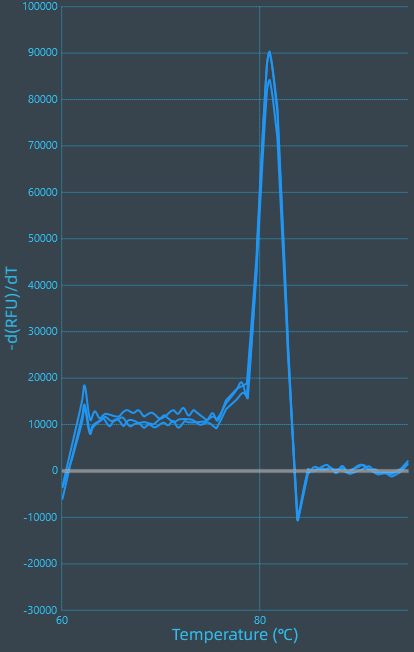


*ZaNF-YA20*
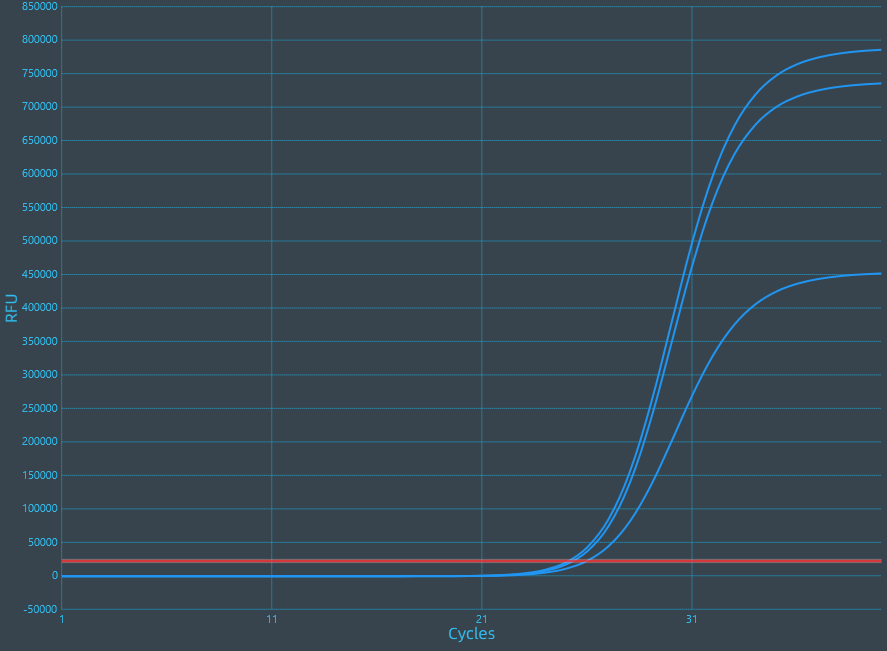

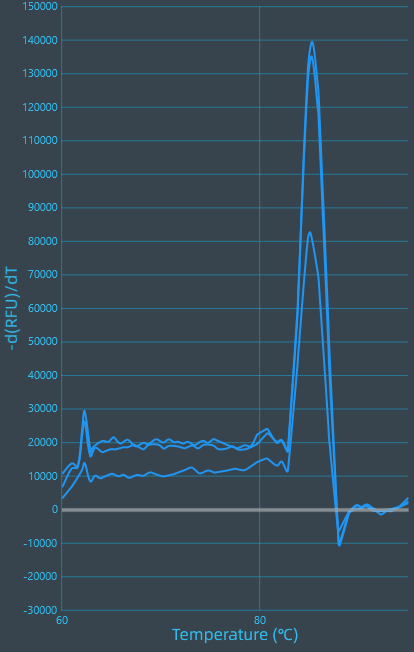


*ZaNF-YB1*
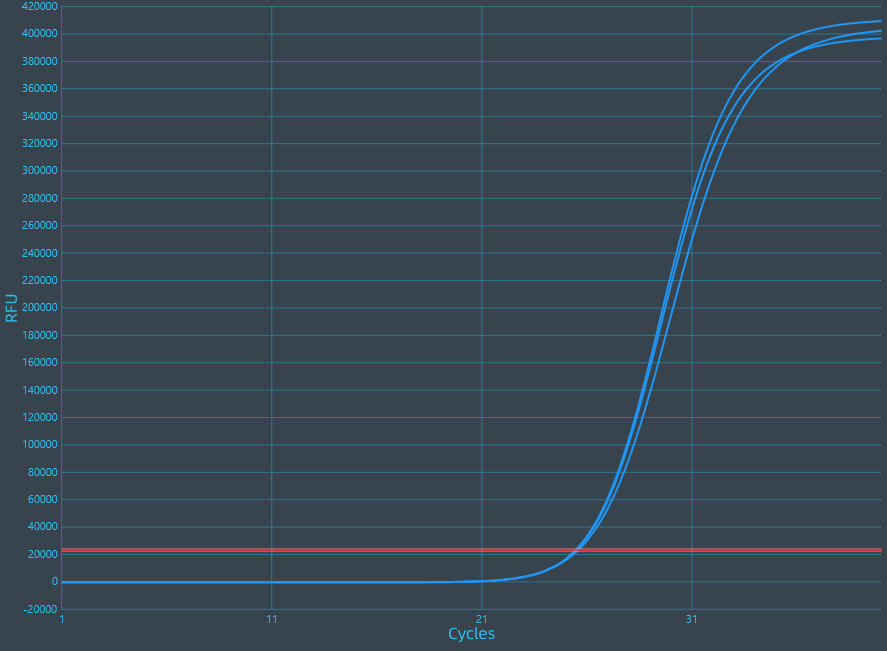

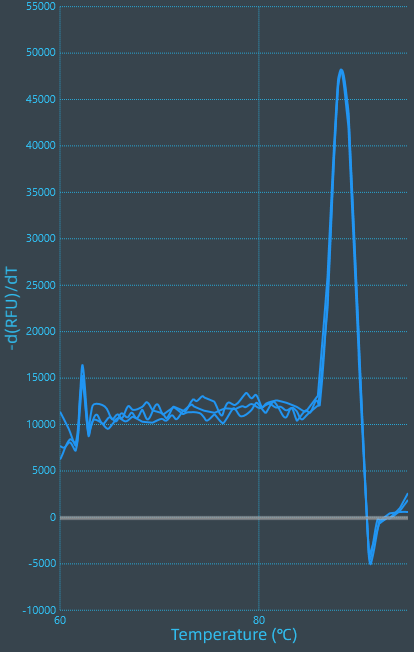


*ZaNF-YB5*
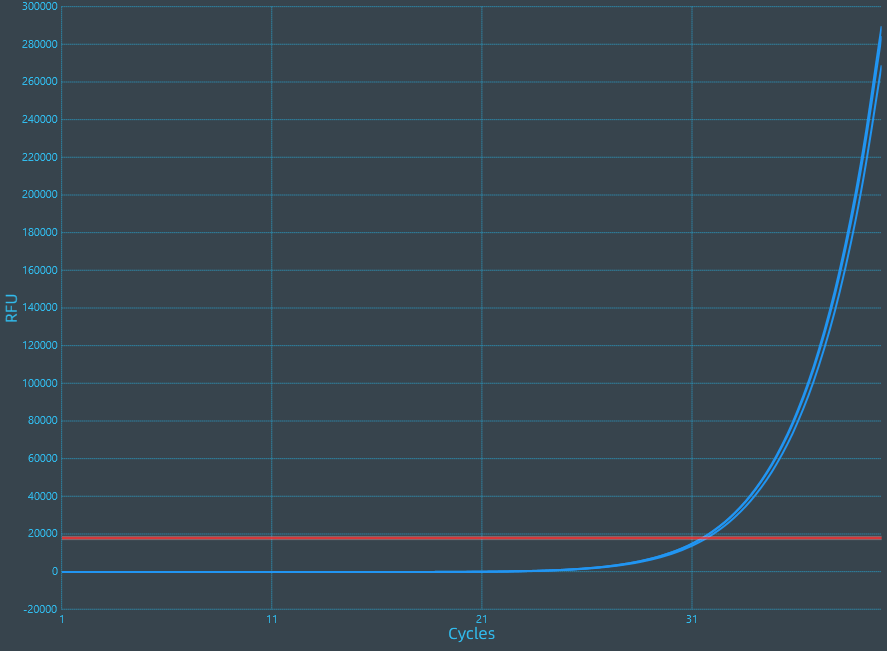

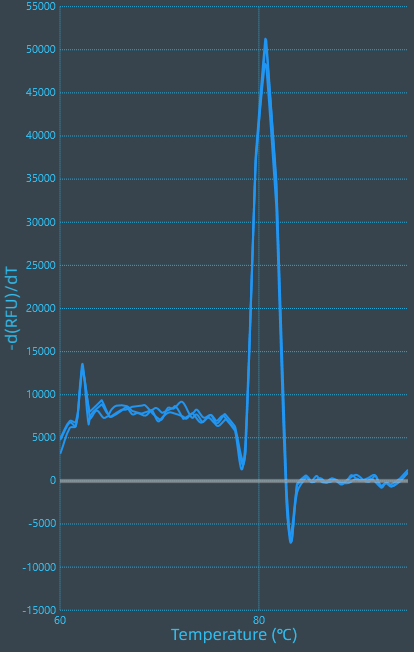


*ZaNF-YB6*
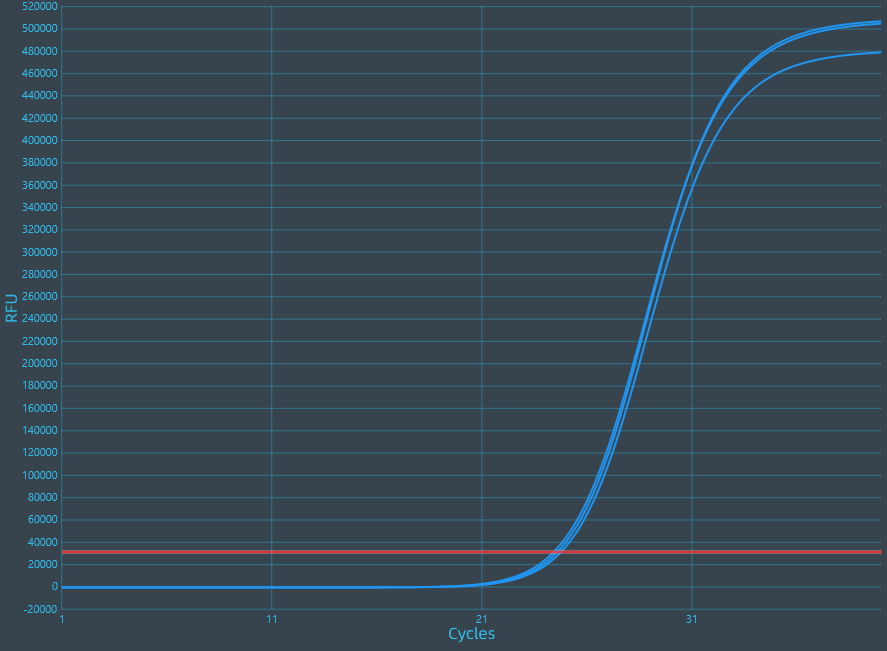

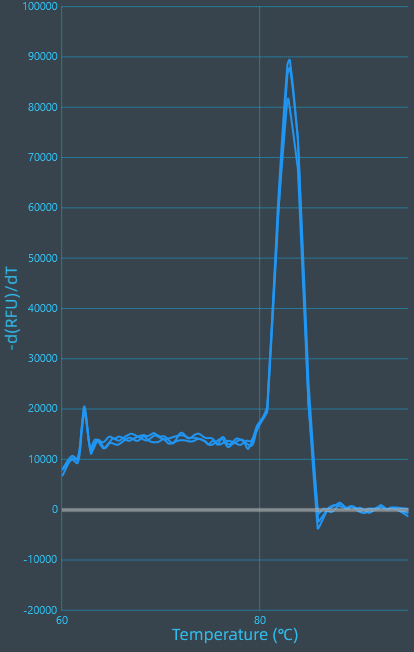


*ZaNF-YB7*
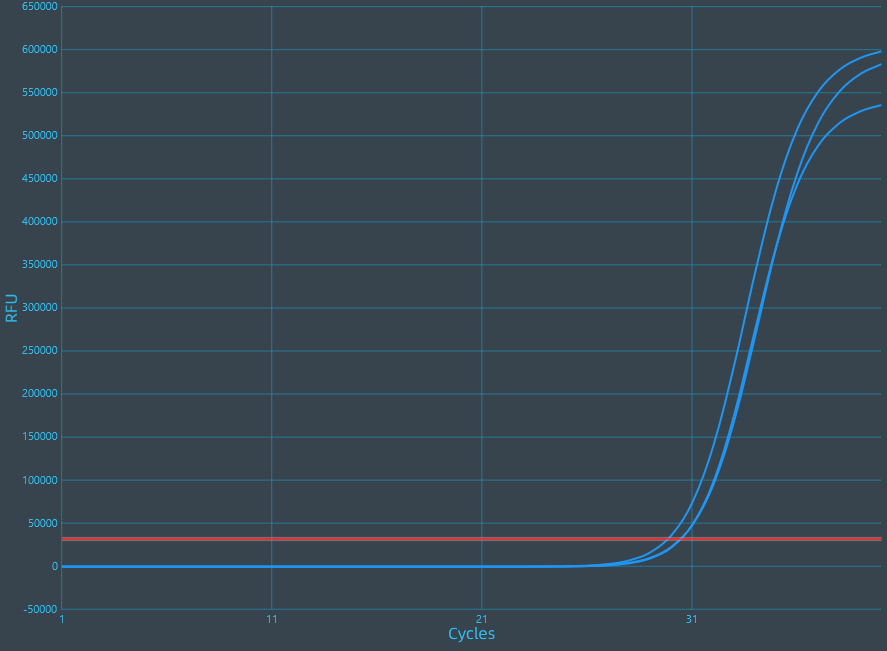

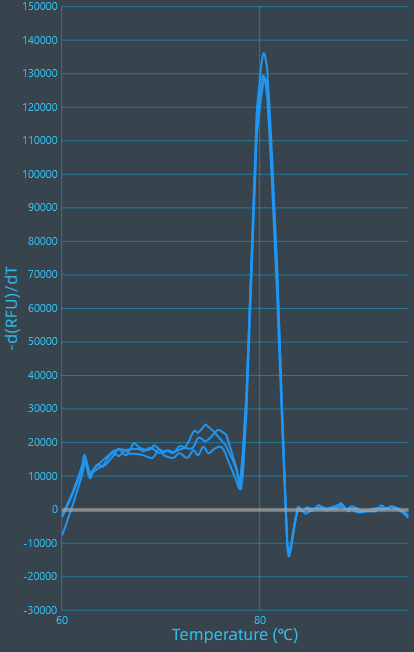


*ZaNF-YB8*
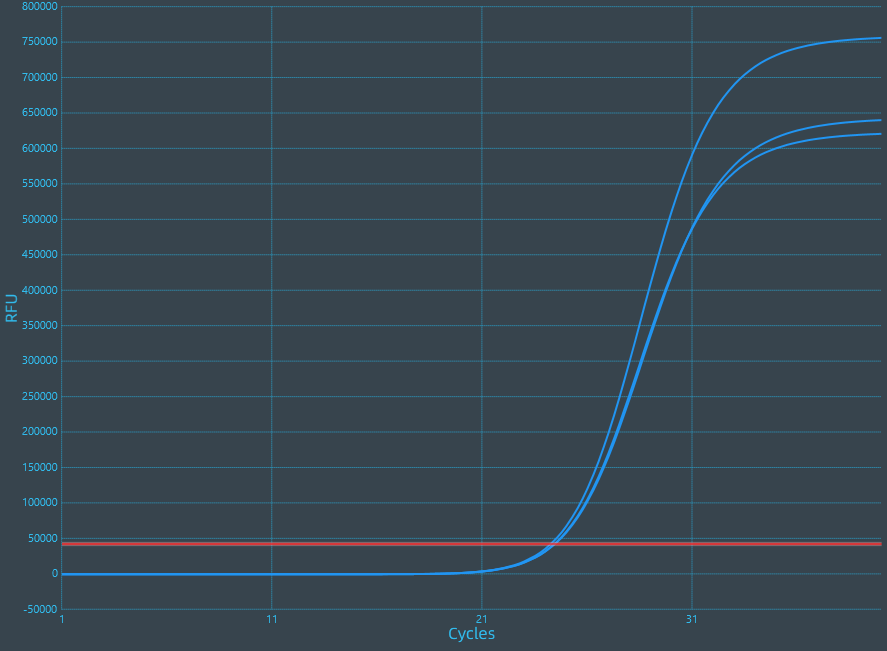

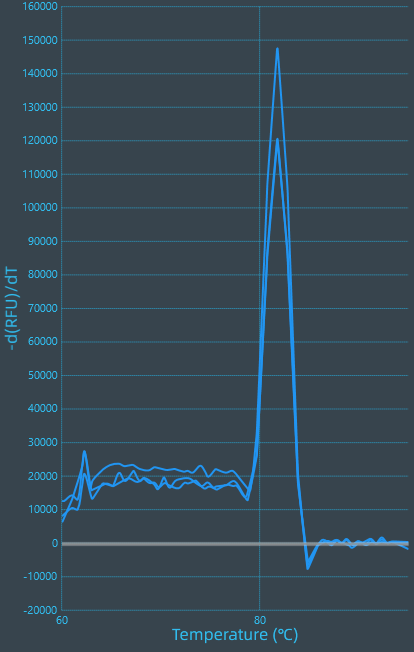


*ZaNF-YB9*
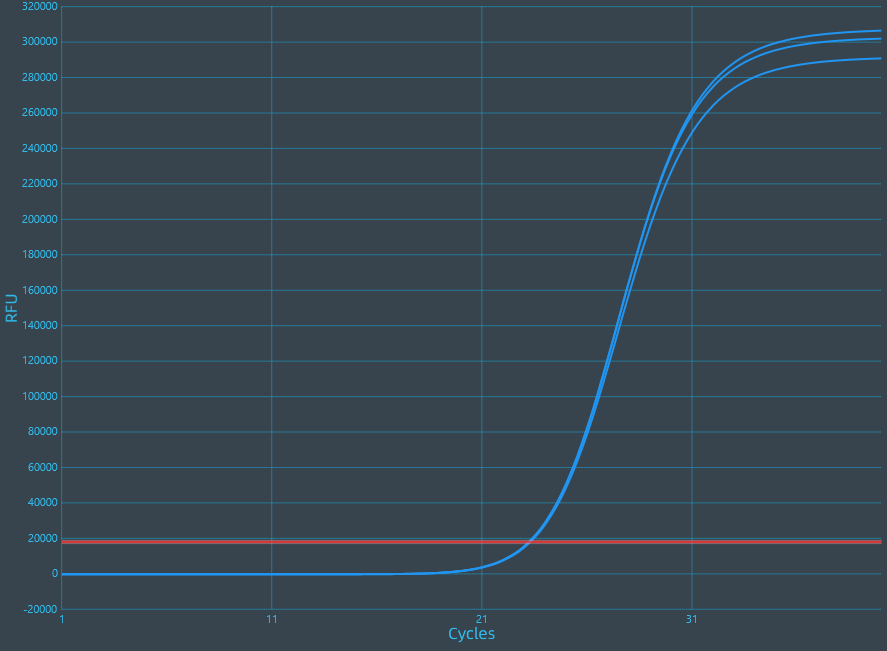

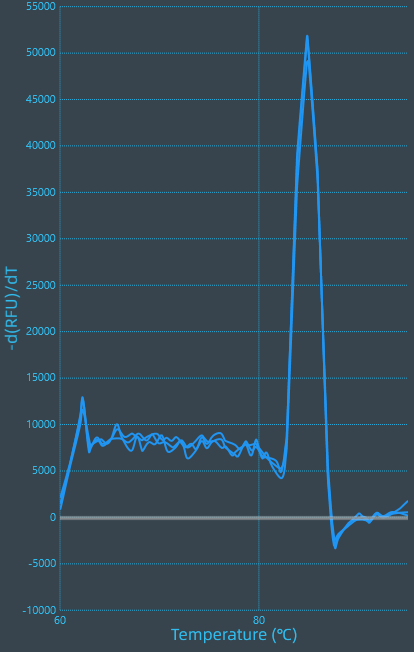


*ZaNF-YB10*
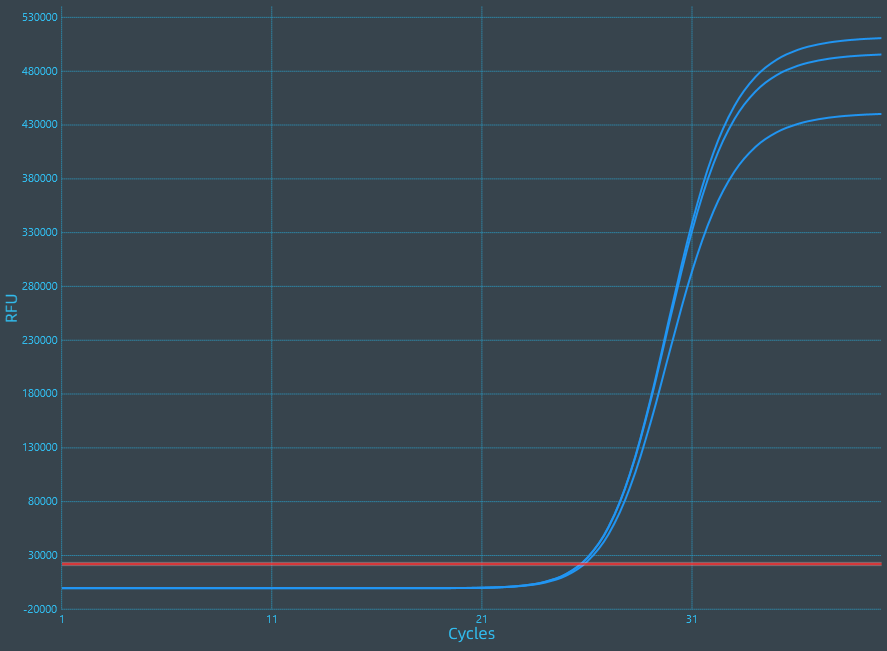

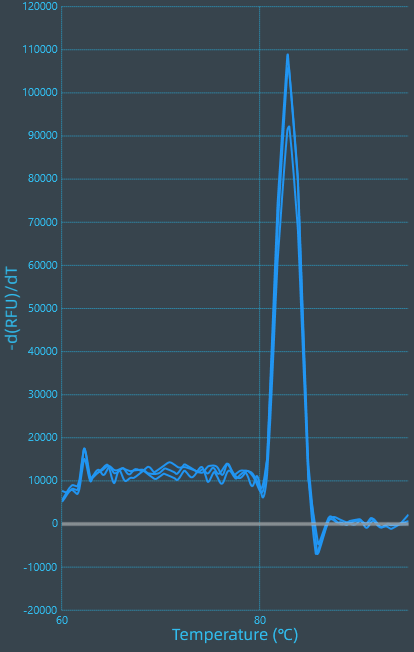


*ZaNF-YB11*
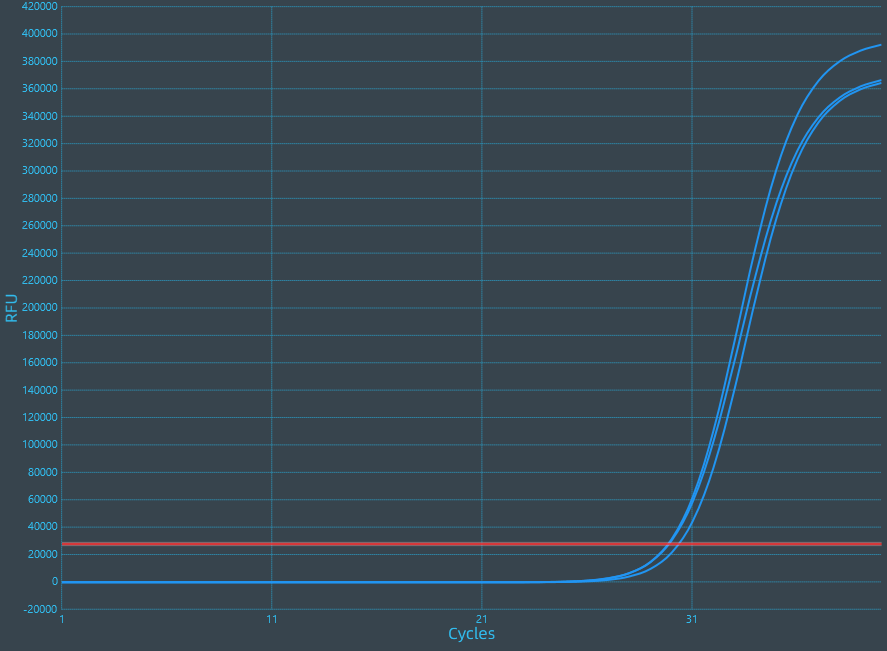

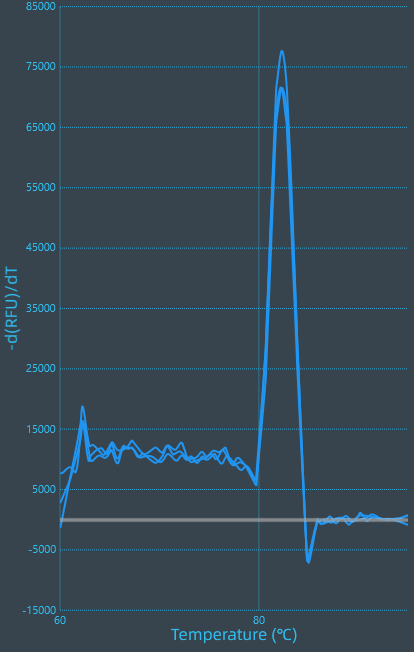


*ZaNF-YB12*
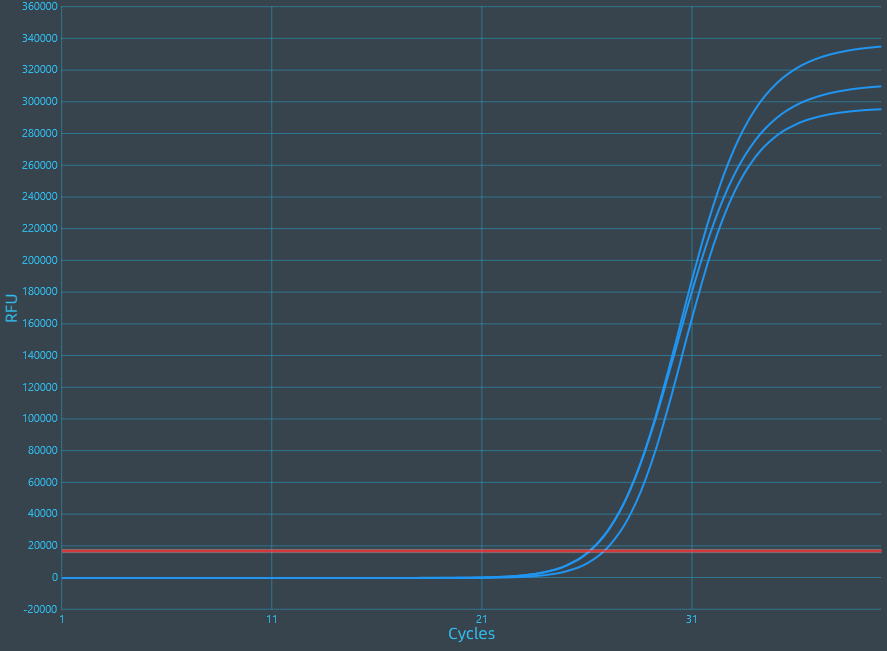

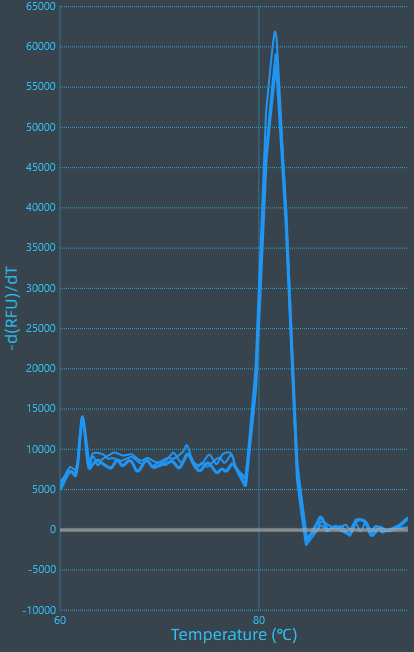


*ZaNF-YB13*
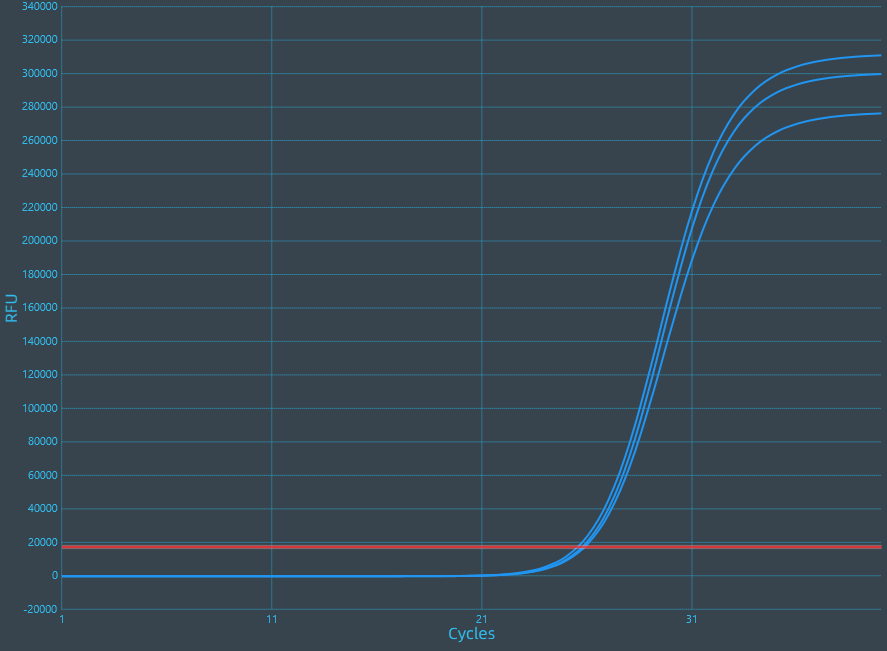

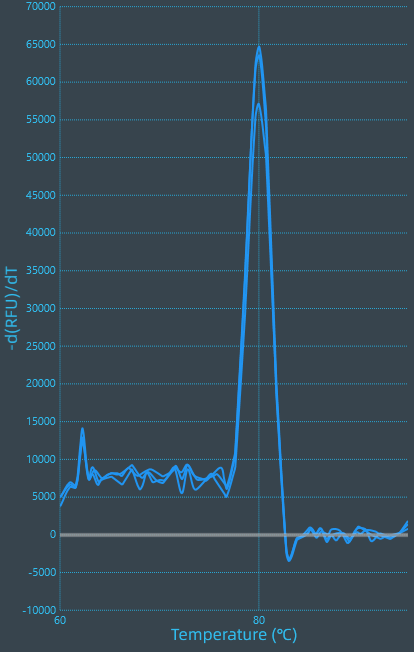


*ZaNF-YB15*
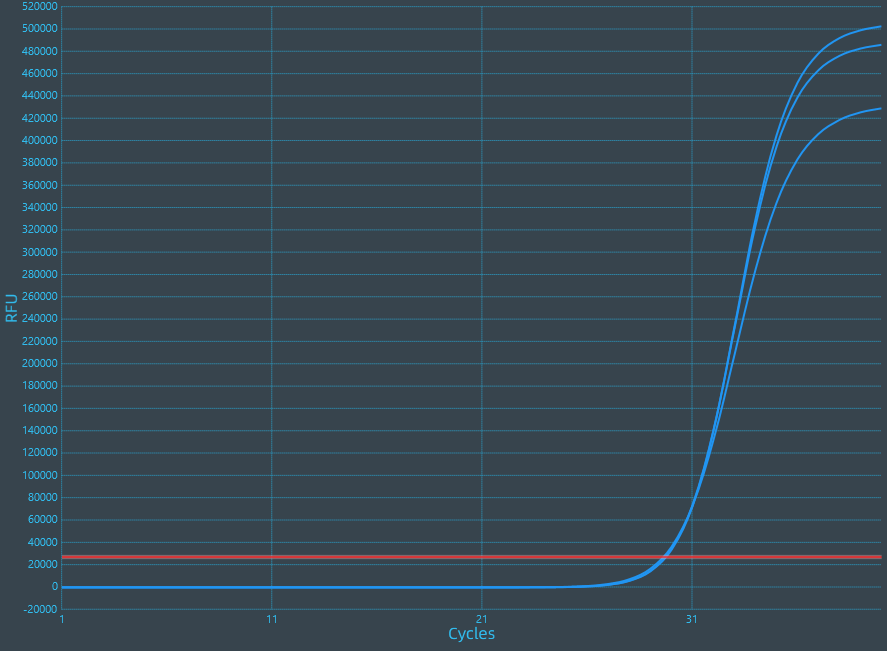

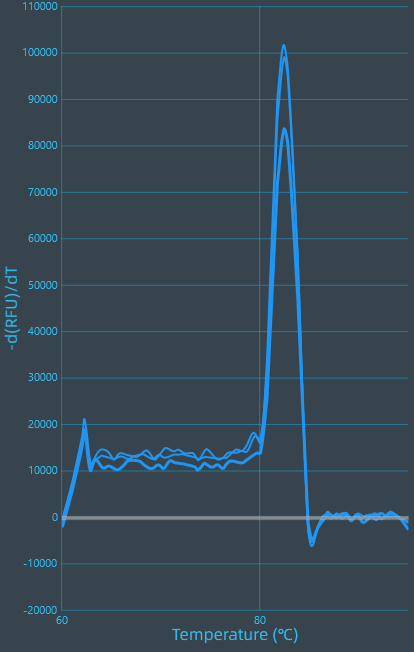


*ZaNF-YB16*
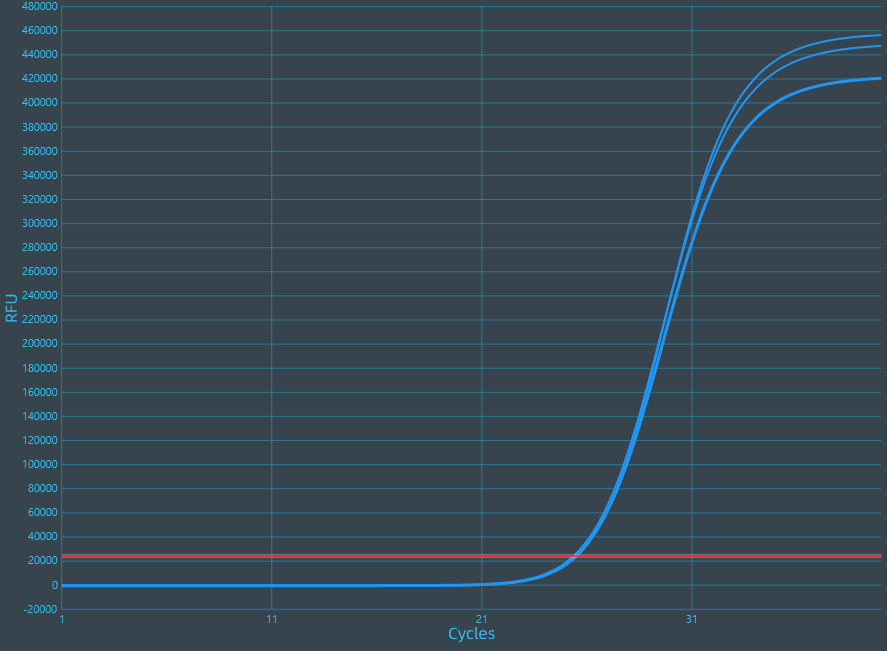

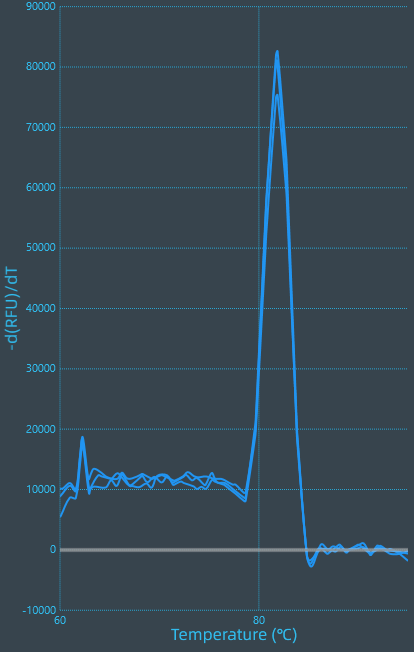


*ZaNF-YB18*
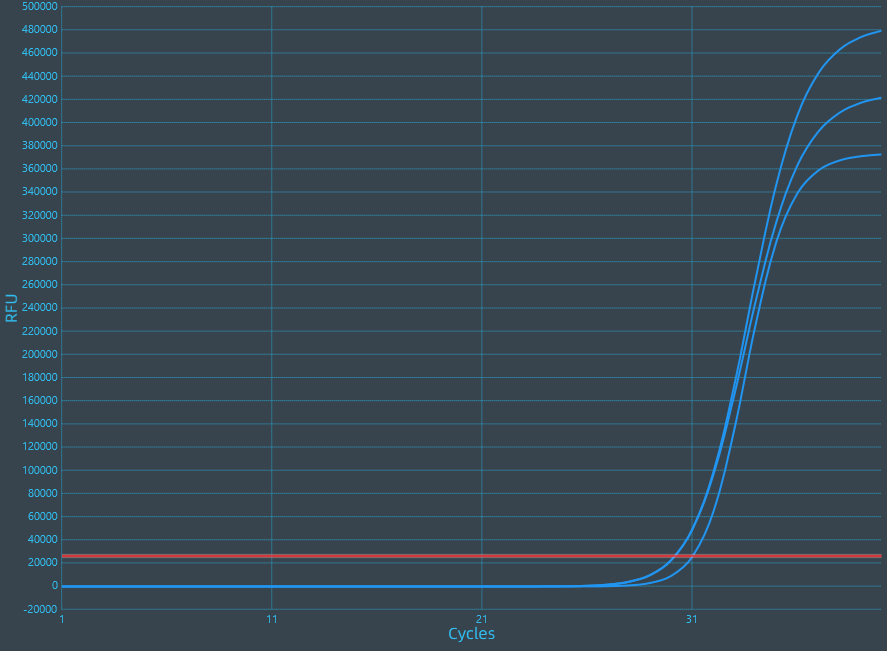

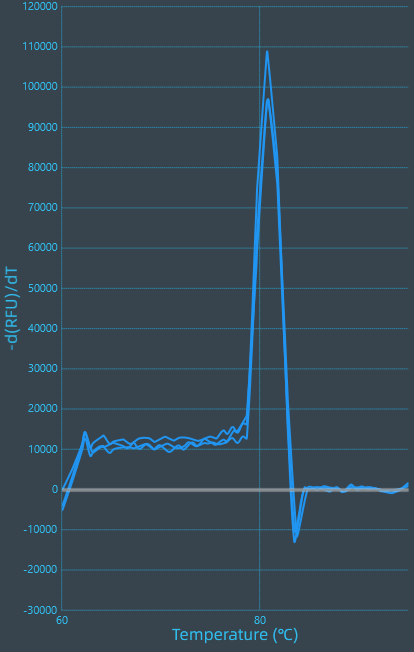


*ZaNF-YB19*
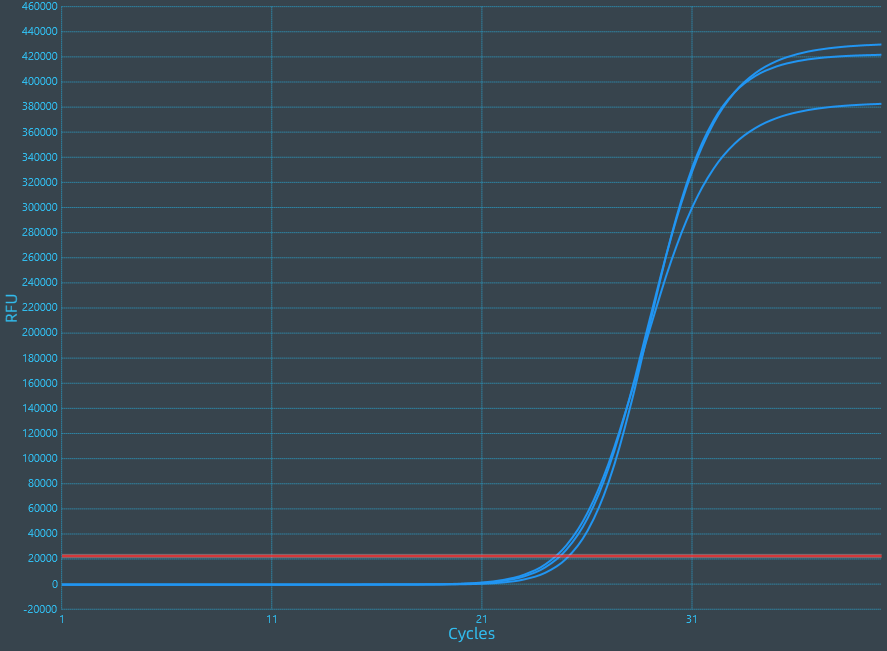

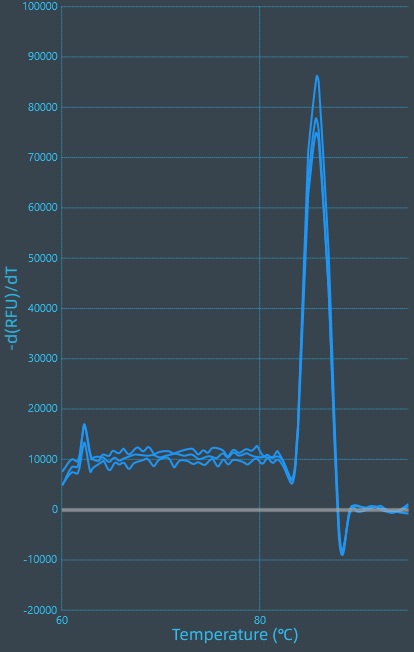


*ZaNF-YB20*
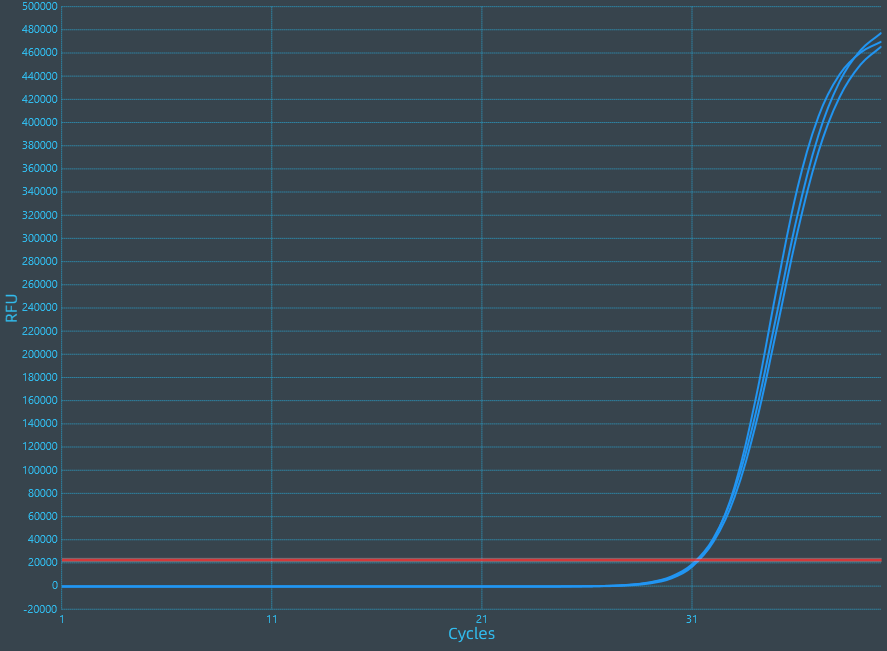

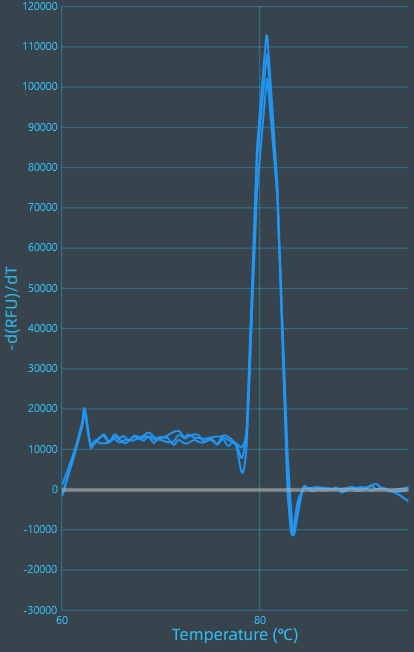


*ZaNF-YB21*
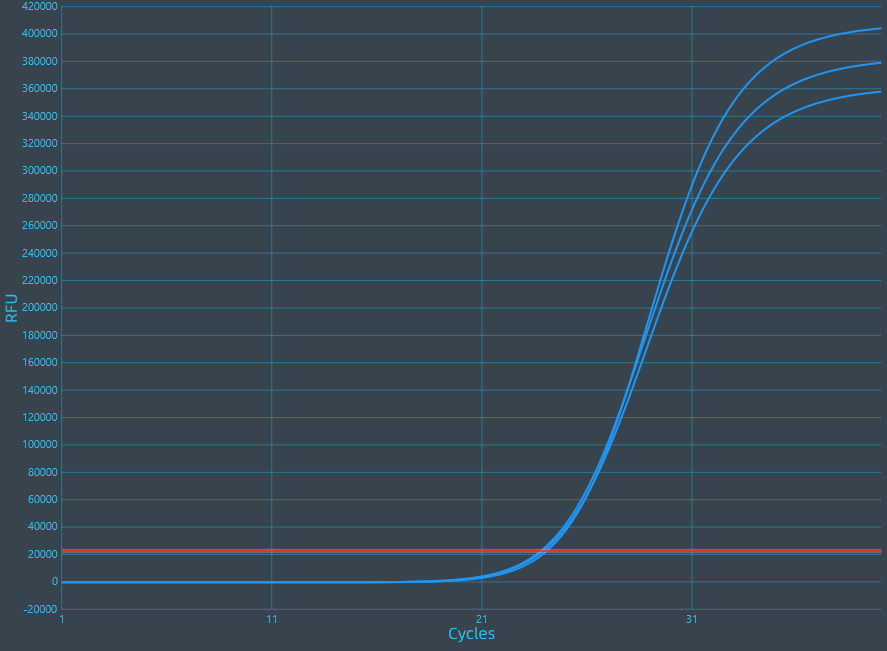

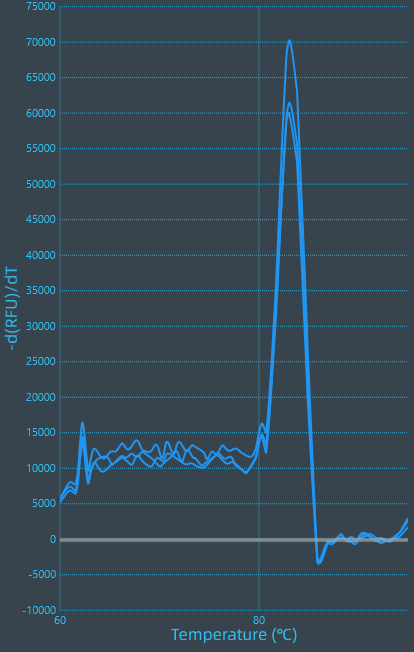


*ZaNF-YB24*
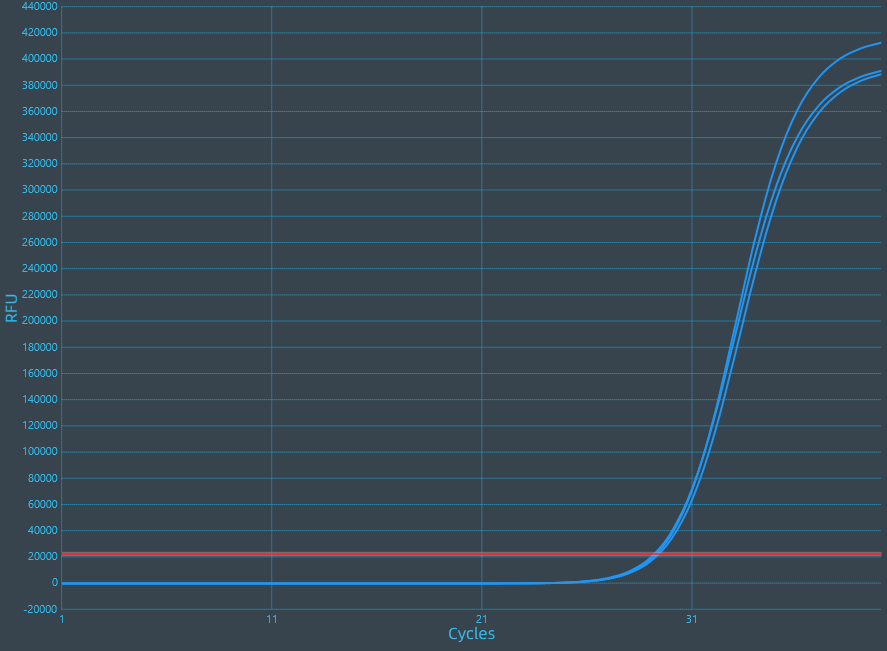

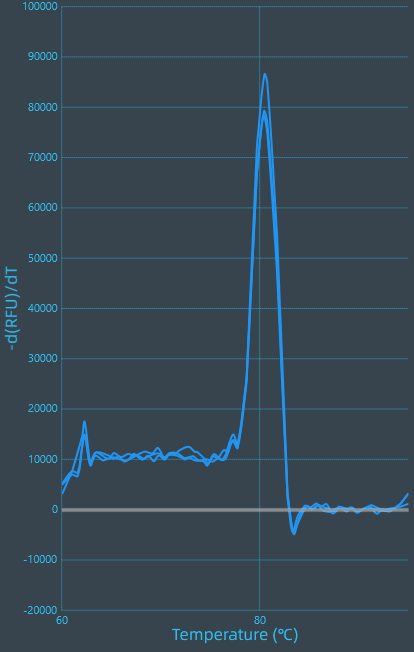


*ZaNF-YB28*
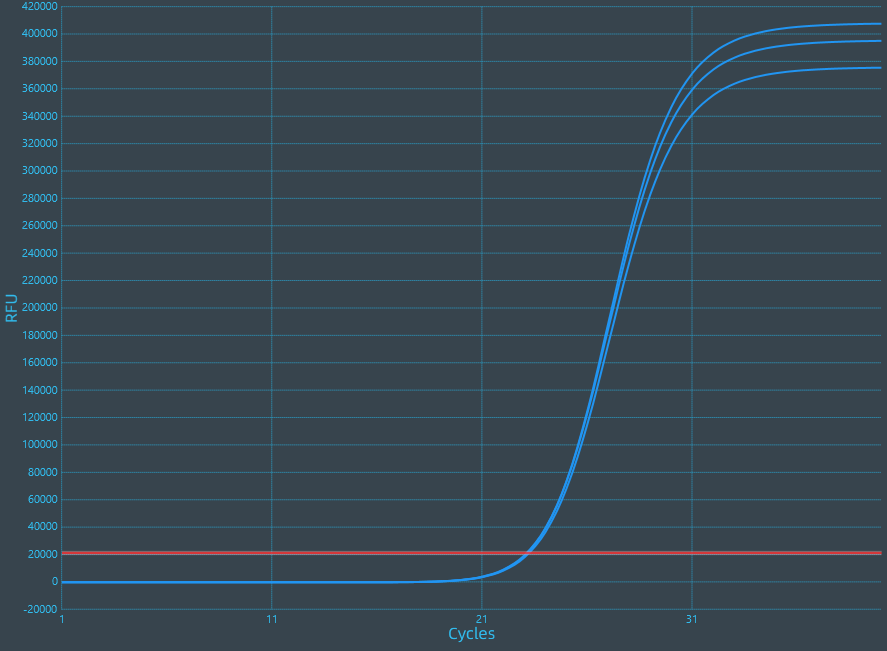

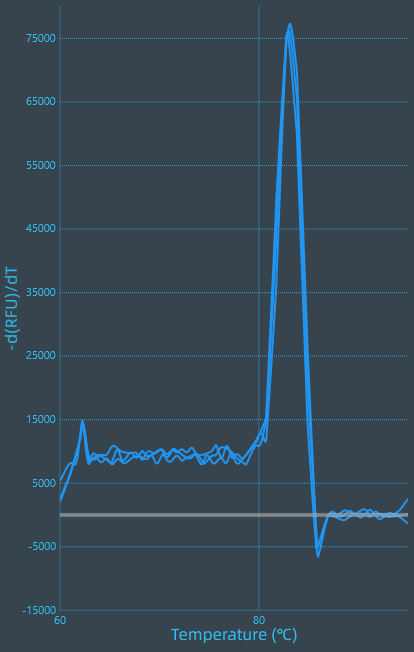


*ZaNF-YB29*
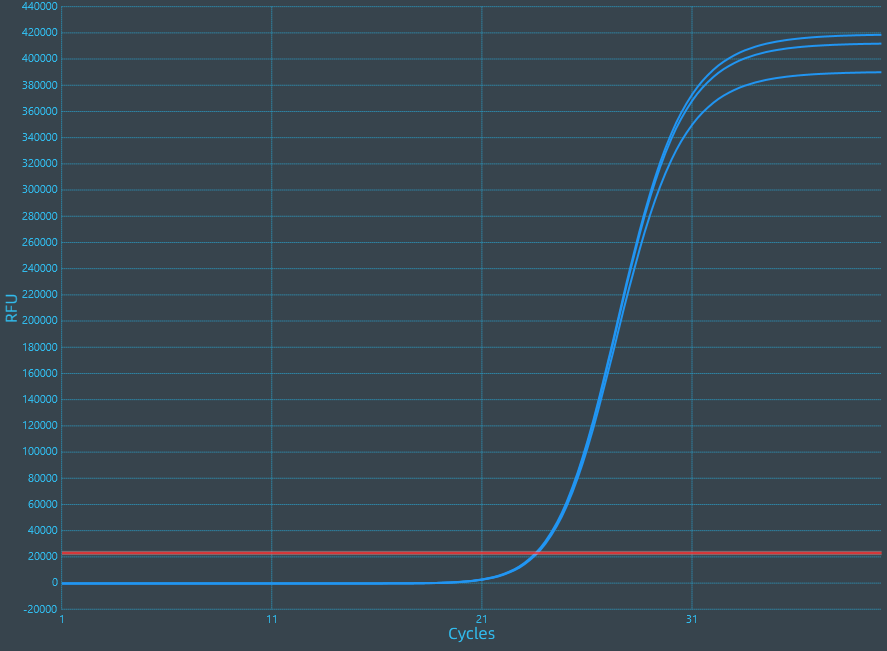

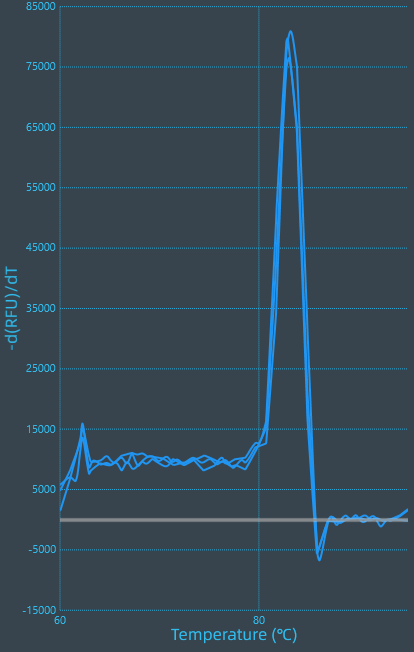


*ZaNF-YC1*
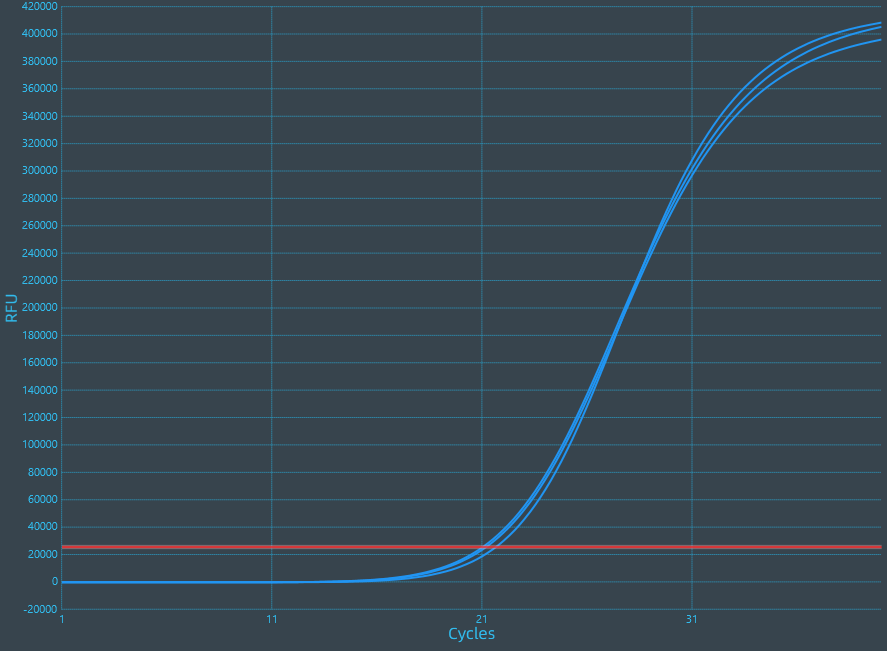

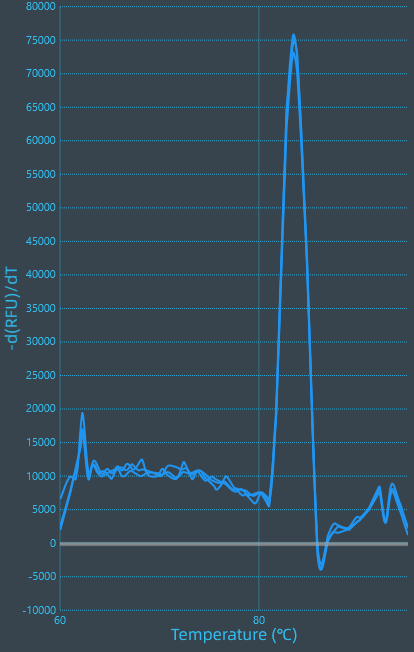


*ZaNF-YC2*
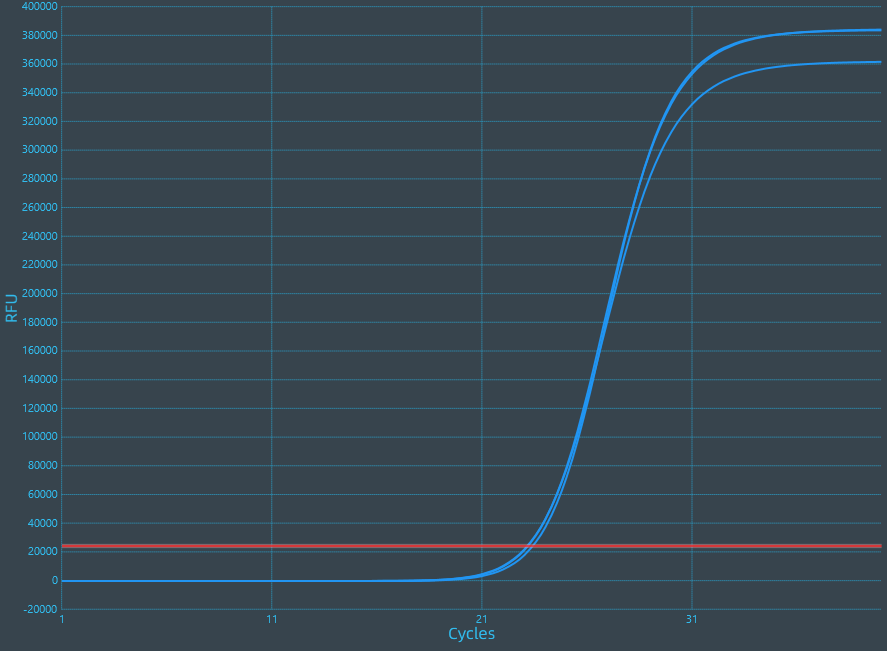

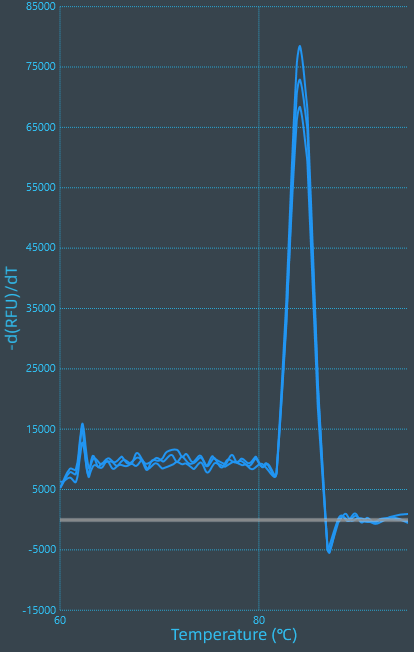


*ZaNF-YC4*
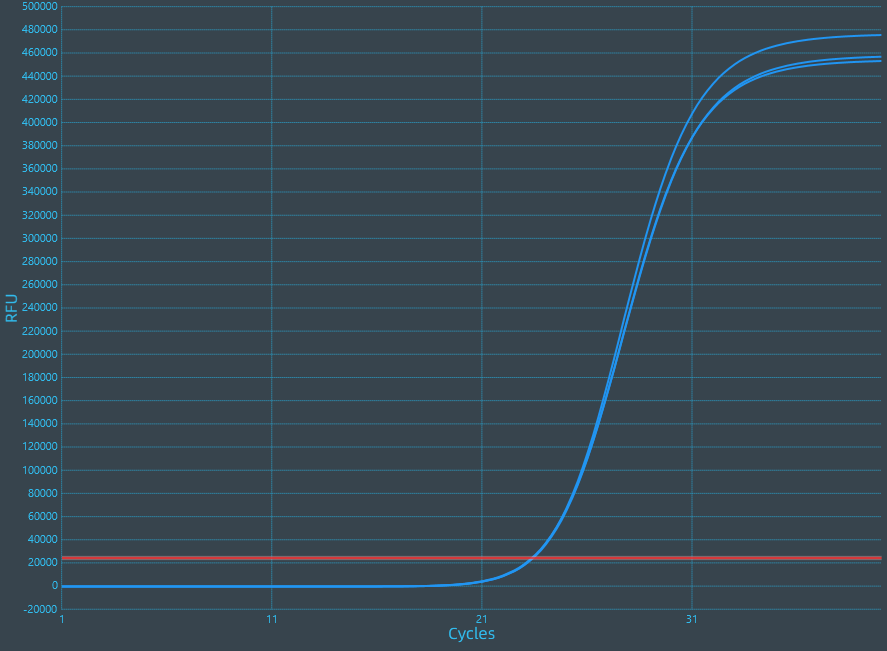

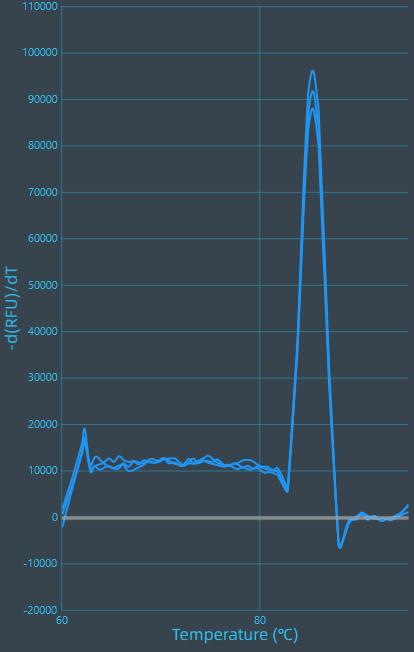


*ZaNF-YC5*
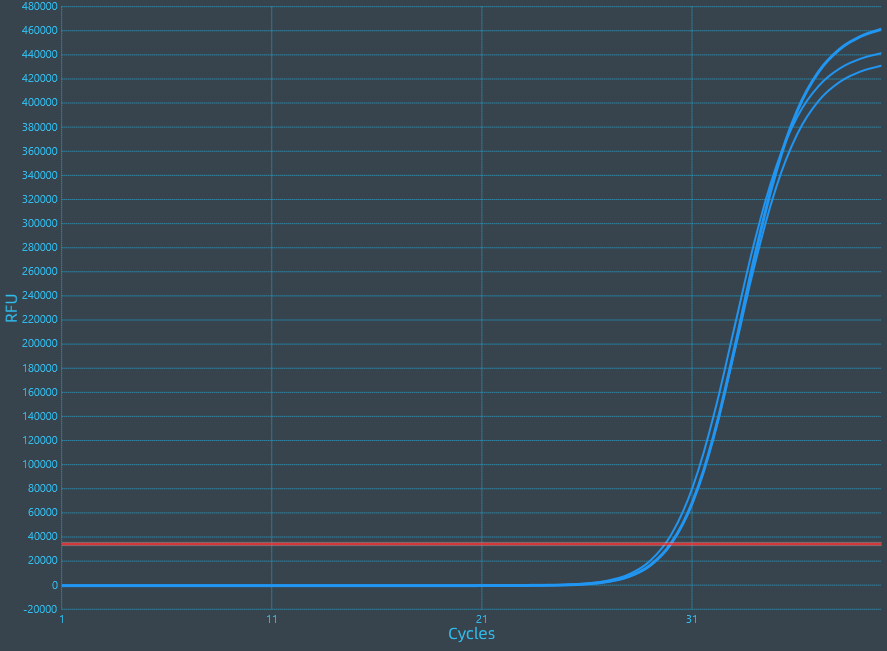

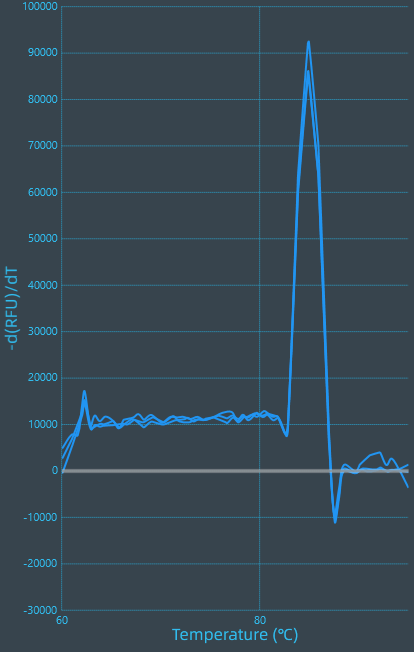


*ZaNF-YC6*
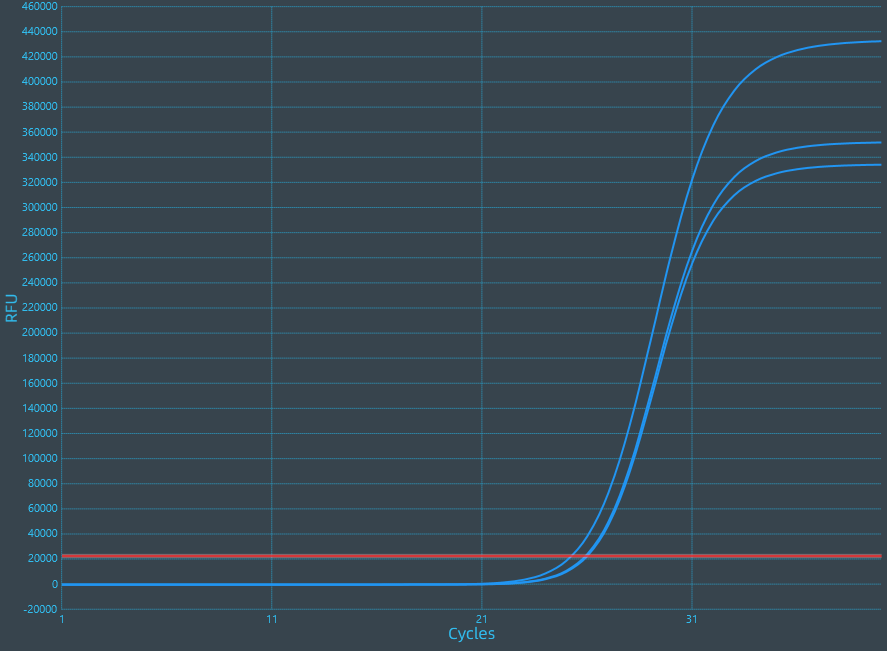

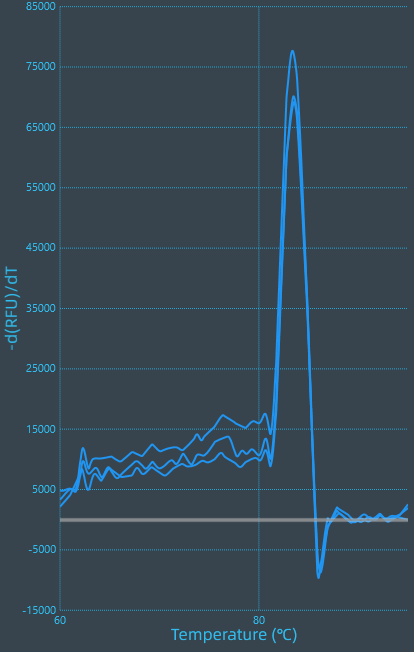


*ZaNF-YC9*
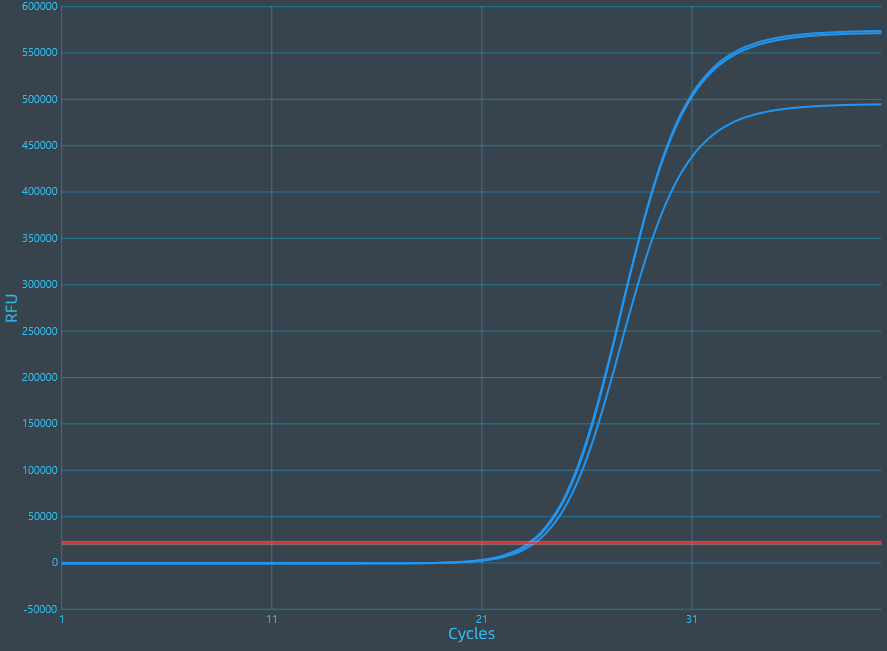

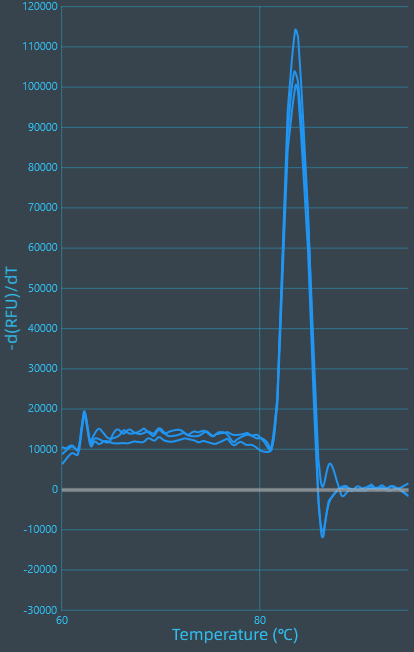


*ZaNF-YC10*
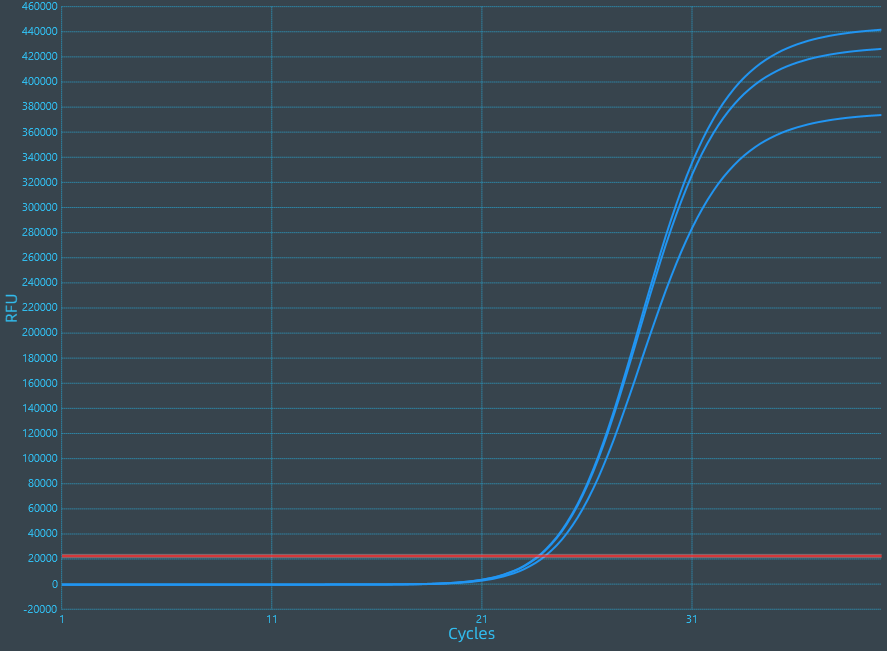

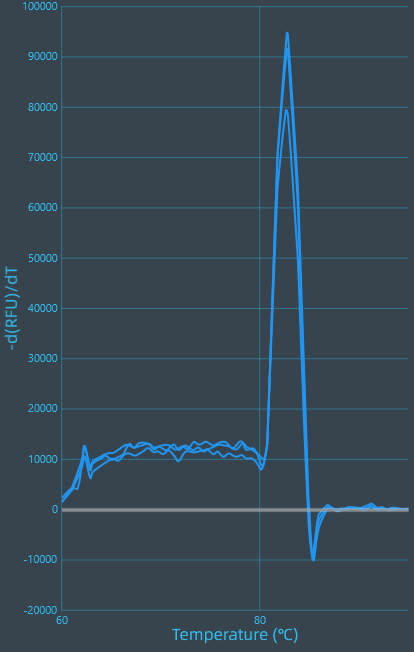


*ZaNF-YC11*
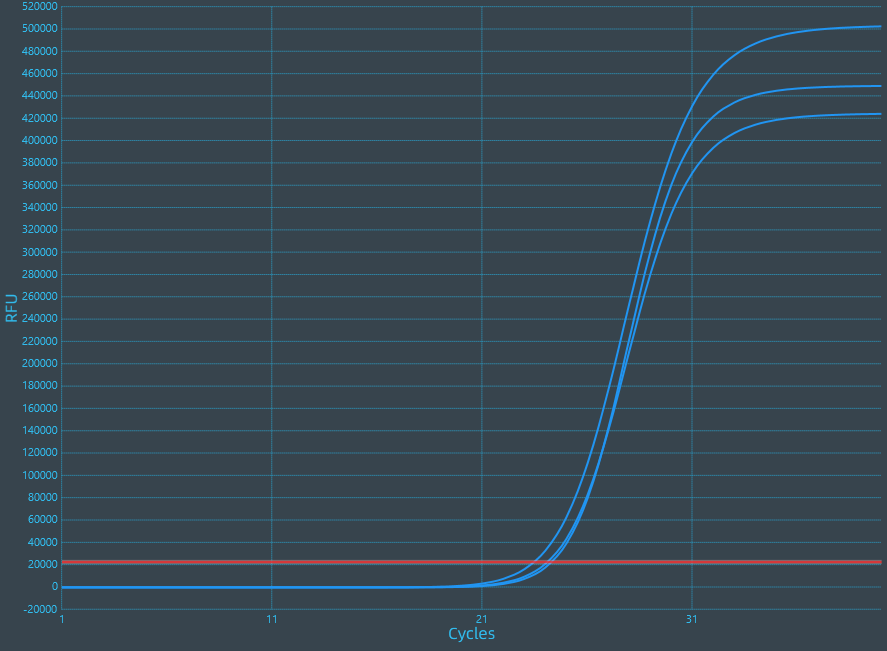

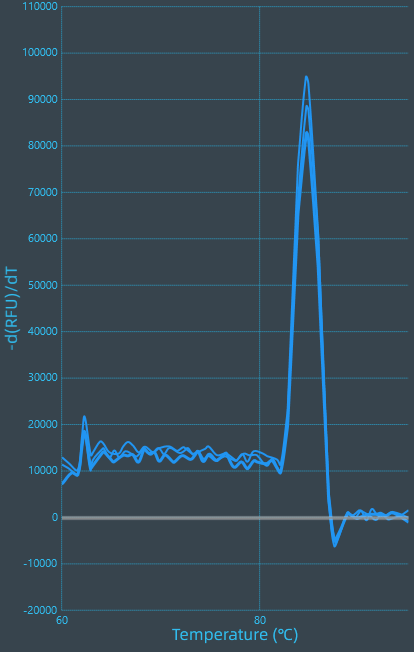


*ZaNF-YC12*
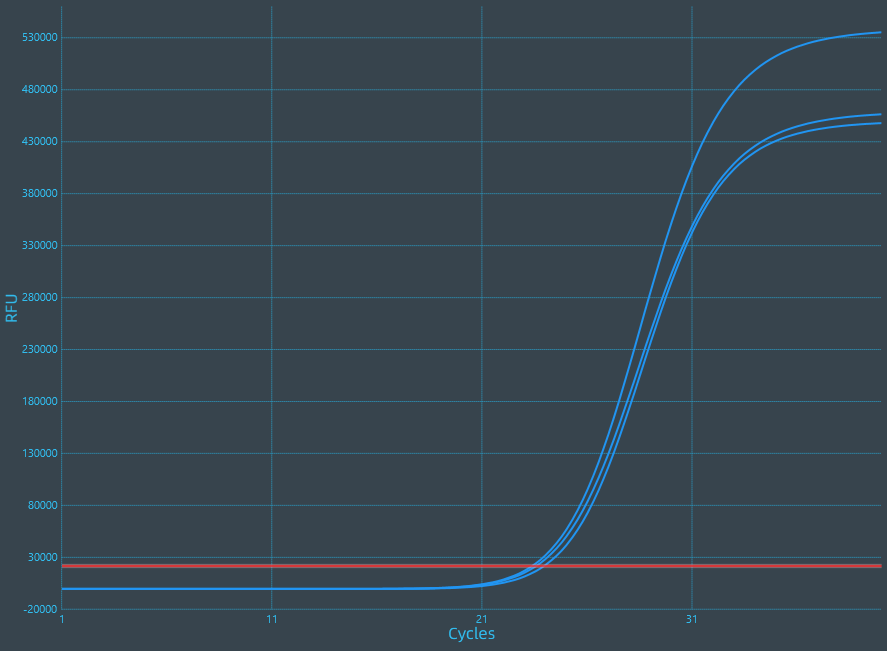

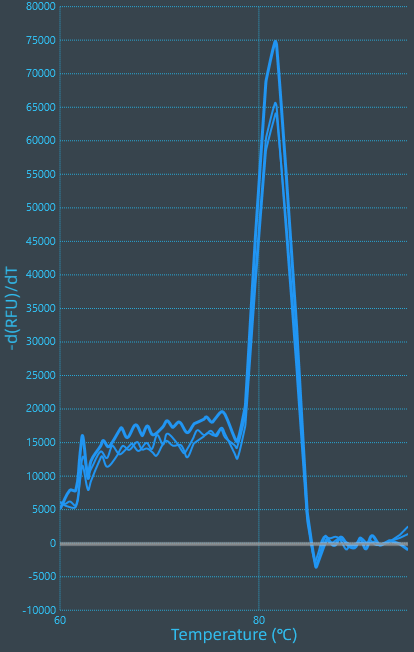


*ZaNF-YC13*
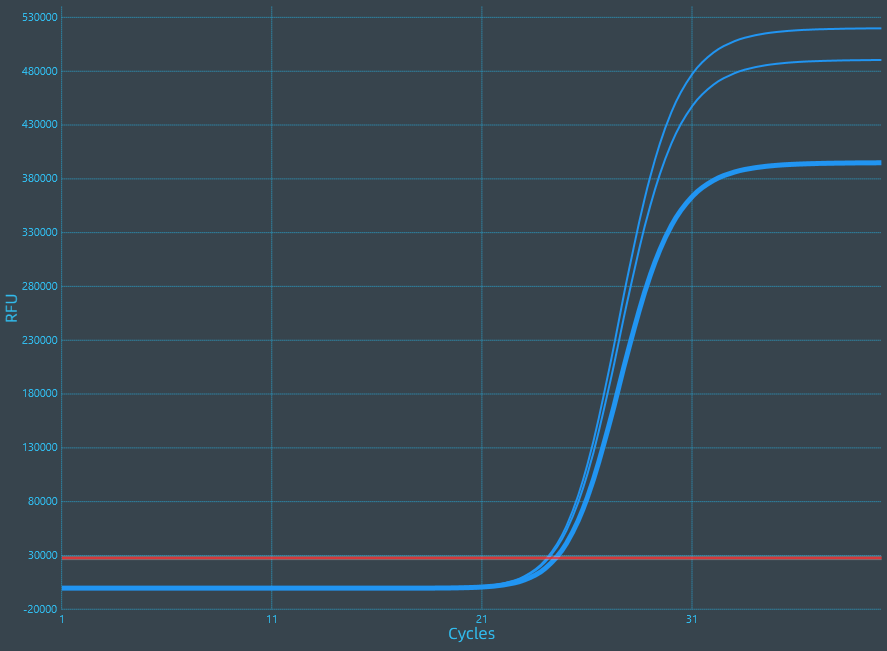

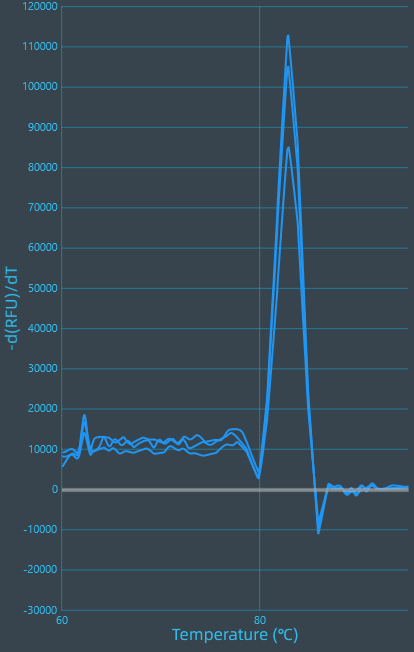


*ZaNF-YC14*
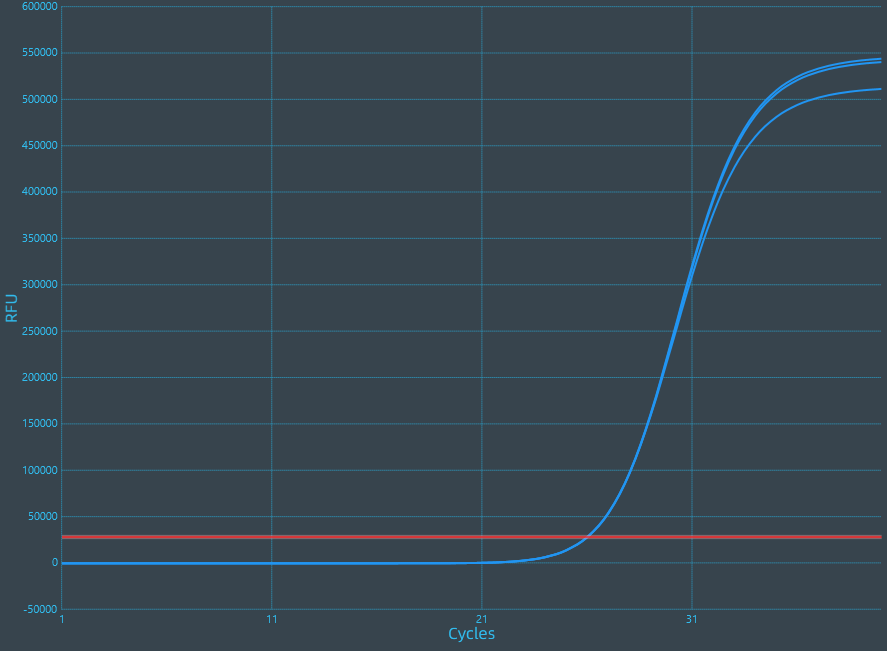

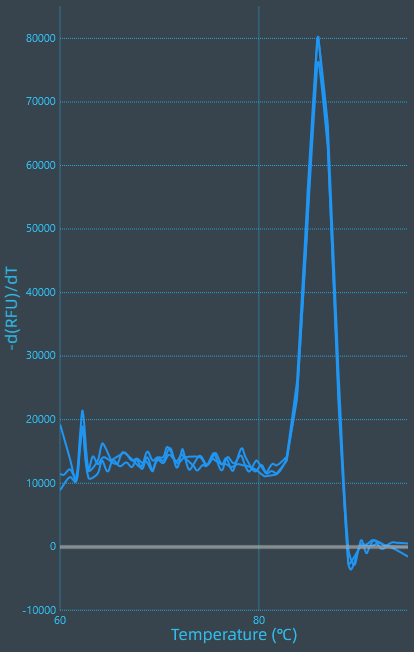


*ZaNF-YC15*
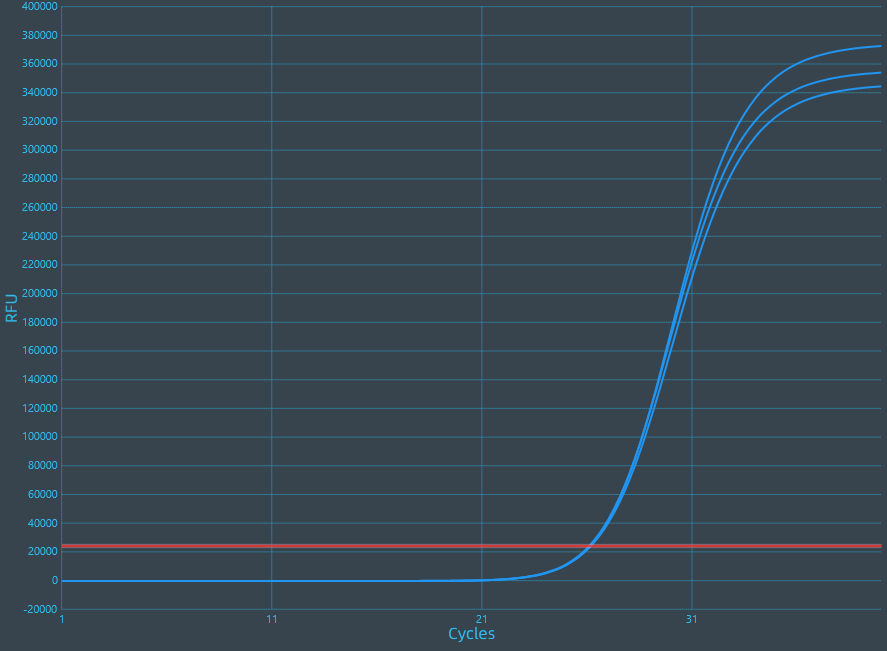

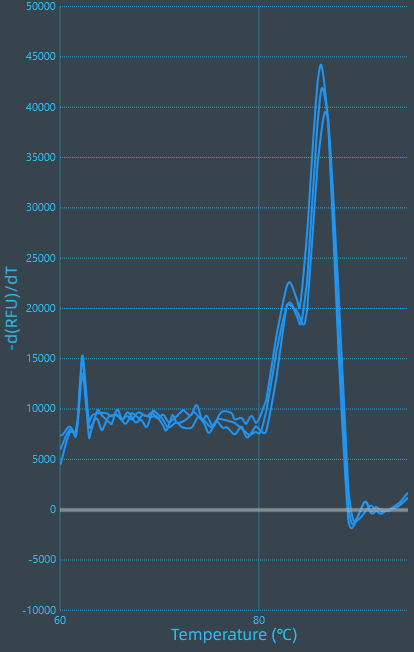


*ZaNF-YC16*
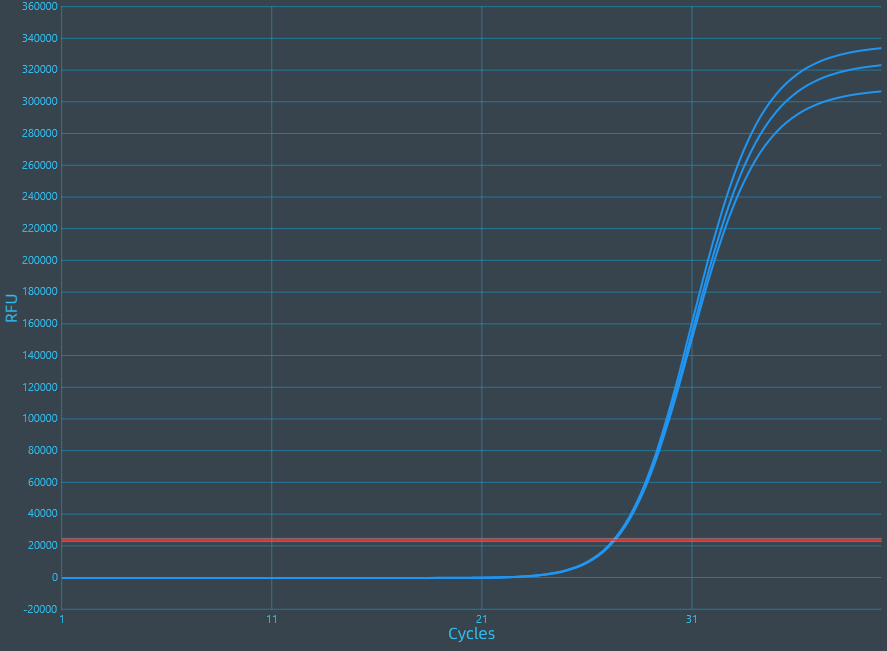

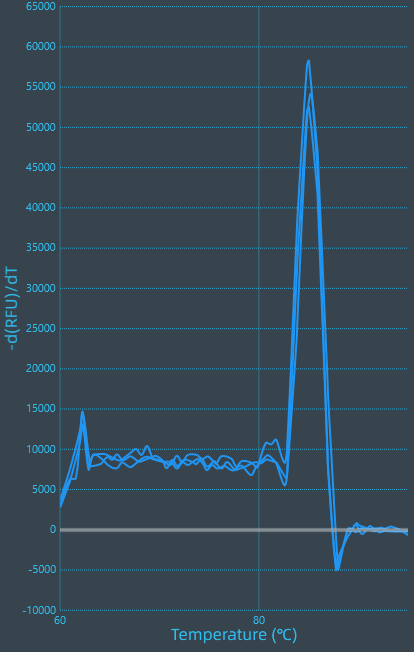


*ZaNF-YC17*
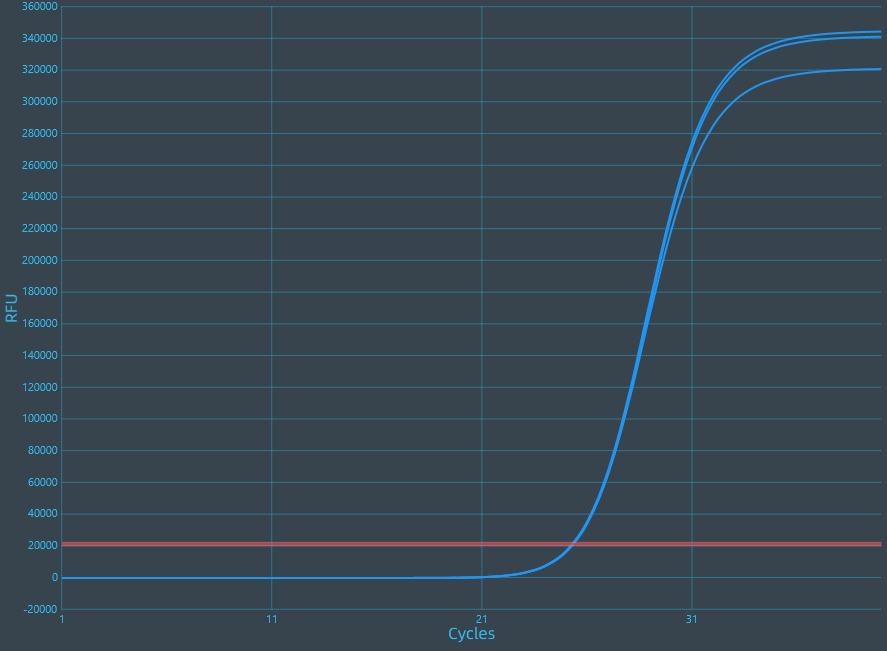

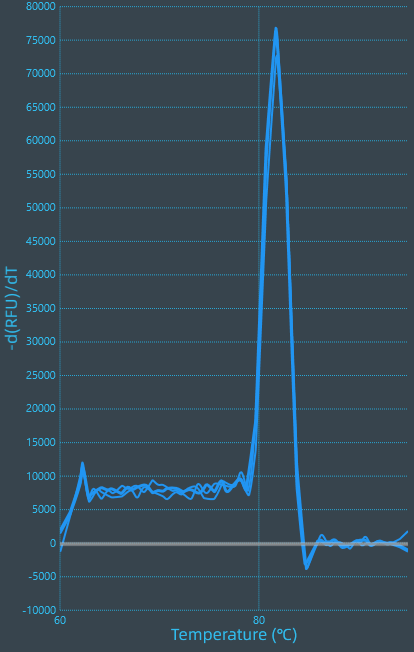


*ZaGAPDH* **
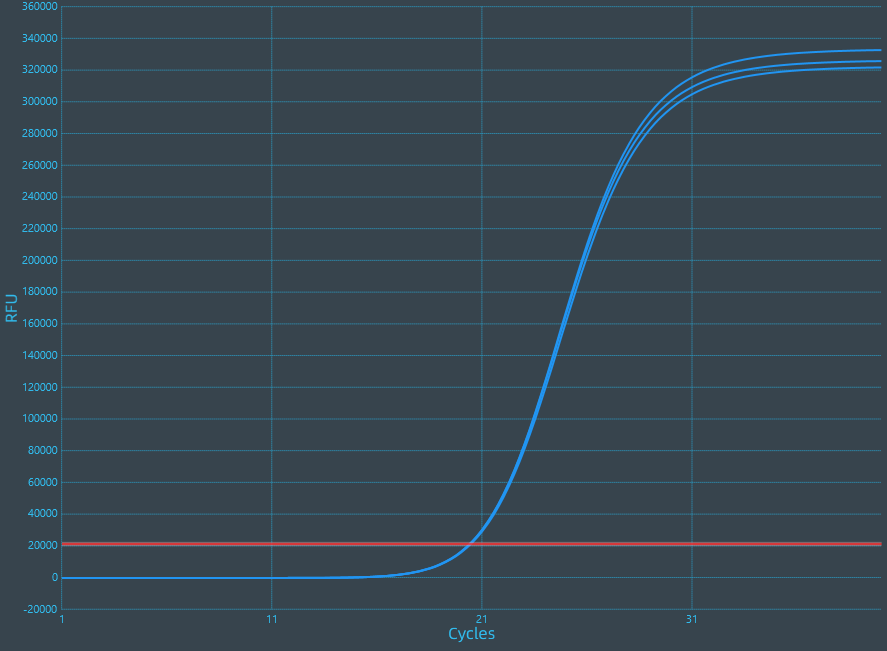
**
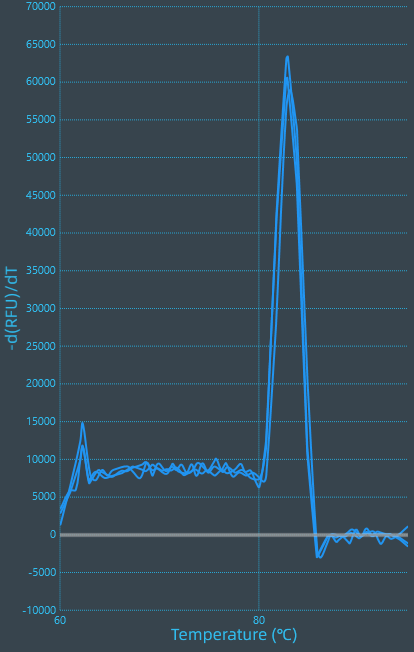


**7.2 Calibration curves and PCR efficiency calculated from slope**

To verify the primer amplification efficiency and create standard curves, a concentration gradient was prepared by serially diluting the cDNA templates with 5-fold dilution to give 1/5, 1/25, 1/125, and 1/625 times dilutions. A standard curve was drawn from the obtained Ct values to obtain the slope k and linear correlation coefficient (regression coefficient) R2. The amplification efficiency E (E = 10^(−1/k)^ − 1) was calculated using the obtained slope k.


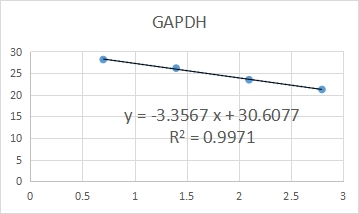


K = -3.3567 Amplification efficiency (E, %) = 98.57%

R^2^ = 0.9971

**7.3 Results for NTCs**

*ZaNF-YA1*

*ZaNF-YA2*

*ZaNF-YA3*

*ZaNF-YA4*

*ZaNF-YA5*

*ZaNF-YA8*

*ZaNF-YA9*

*ZaNF-YA11*

*ZaNF-YA12*

*ZaNF-YA15*

*ZaNF-YA16*

*ZaNF-YA17*

*ZaNF-YA18*

*ZaNF-YA19*

*ZaNF-YA20*

*ZaNF-YB1*

*ZaNF-YB5*

*ZaNF-YB6*

*ZaNF-YB7*

*ZaNF-YB8*

*ZaNF-YB9*

*ZaNF-YB10*

*ZaNF-YB11*

*ZaNF-YB12*

*ZaNF-YB13*

*ZaNF-YB15*

*ZaNF-YB16*

*ZaNF-YB18*

*ZaNF-YB19*

*ZaNF-YB20*

*ZaNF-YB21*

*ZaNF-YB24*

*ZaNF-YB28*

*ZaNF-YB29*

*ZaNF-YC1*

*ZaNF-YC2*

*ZaNF-YC4*

*ZaNF-YC5*

*ZaNF-YC6*

*ZaNF-YC9*

*ZaNF-YC10*

*ZaNF-YC11*

*ZaNF-YC12*

*ZaNF-YC13*

*ZaNF-YC14*

*ZaNF-YC15*

*ZaNF-YC16*

*ZaNF-YC17*

*ZaGAPDH*

**8 Data analysis**

**8.1 qPCR analysis program (source, version)**

JLM-qPCR-AnalyserSoft V1.4.4.9.

**8.1 Cq method determination**

The Cq value range is between 15-35.

**8.1 Outlier identification and disposition**

The Cq exceeding 35 indicates that it has not been amplified.If the peak starts before 15, it may caused by the high template concentration, we should changed the template concentration.

**8.2 Statistical methods for results significance**

One-WayANOVA（LSD）

**8.3 Software (source, version)**

International Business Machines Statistical Package for the Social Sciences software (version 27.0)

**8.4 Justification of number and choice of reference genes**

In previous research, 13 reference genes (*GAPDH, GAPDA1, GAPDA2, ACT, TUB1, TUB9, EF2-2, EF2-3, EF2-4, ELOF1, EF1B1, eIF6-2*) were screened according to transcriptome data, and their stability in different tissues (stems, leaves, flowers, fruits, petioles, and calluses) were analyzed using four algorithms: geNorm, NormFinder, BestKeeper, and RefFinder. The results showed that *GAPDH*, *EF2-4,* and *eIF6-2* were stably expressed in different tissues. The research results have not yet been published.

**8.5 Description of normalization method**

Relative quantitative analysis of *ZaNF-Y* genes in different tissues were calculated using the 2^–ΔCt^ method, the expression level was log_2_ transformed and normalized to obtain a heatmap by TBtools.
